# Xinlikang Capsule Alleviates Chemotherapy‐Induced Fatigue by Inhibiting the PI3K/AKT‐mTOR‐FoxO Pathway

**DOI:** 10.1155/ancp/8879160

**Published:** 2026-02-19

**Authors:** Suzhou Huang, Yiheng Zhang, Tianle Ma, Lu Zhang, Xingxing Lu, Huihua Fang, Gaohong Lv, Zhipeng Chen, Xin Liu, Lina Zhang, Li Wu

**Affiliations:** ^1^ Jiangsu Key Laboratory for Pharmacology and Safety Research of Chinese Materia Medica, School of Pharmacy, Nanjing University of Chinese Medicine, Nanjing, China, njucm.edu.cn; ^2^ The Second Medical Department, Shanghai Haitian Pharmaceutical Technology Development Co., Ltd., Shanghai, China; ^3^ Jiangsu Province Hospital of Chinese Medicine, Affiliated Hospital of Nanjing University of Chinese Medicine, Nanjing, China, njucm.edu.cn; ^4^ Engineering Center of State Ministry of Education for Standardization of Chinese Medicine Processing, School of Pharmacy, Nanjing University of Chinese Medicine, Nanjing, China, njucm.edu.cn; ^5^ Department of Pharmacy, The Affiliated Suzhou Hospital of Nanjing Medical University, Suzhou, China, njmu.edu.cn

**Keywords:** chemotherapy-induced fatigue (CIF), glycogen storage, lactate metabolism, network pharmacology, PI3K/AKT-mTOR-FoxO pathway, Xinlikang (XLK) capsule

## Abstract

**Objective:**

Chemotherapy‐induced fatigue (CIF) remains a clinically challenging condition with limited therapeutic options. This study aimed to elucidate the therapeutic potential and underlying mechanisms of the multiherbal Xinlikang (XLK) capsule against CIF using an integrated strategy that combined network pharmacology prediction with experimental validation.

**Methods:**

A murine CIF model was established using 5‐fluorouracil (5‐FU). XLK was administered at various doses to evaluate its efficacy through comprehensive assessments, including behavioral tests (weight‐bearing swimming, tail suspension, and grip strength), histopathology (hematoxylin–eosin [H&E] and periodic acid‐Schiff [PAS] staining), and metabolic indices (lactate and ATP levels). To investigate the mechanisms, an integrated network pharmacology approach was employed to identify bioactive components of XLK, predict their potential targets, and construct a “component‐target‐pathway” network. Core signaling pathways implicated in CIF were prioritized via protein–protein interaction (PPI) and KEGG enrichment analyses. Key predictions were subsequently verified by Western blot analysis.

**Results:**

XLK treatment significantly ameliorated fatigue‐like behaviors, improved muscle glycogen storage, and restored lactate and ATP homeostasis in CIF mice (all *p*  < 0.05). Network pharmacology predicted that the anti‐CIF effect of XLK was closely associated with the regulation of energy metabolism‐related pathways, particularly the PI3K/AKT‐mTOR‐FoxO signaling axis. Experimental validation confirmed that XLK significantly modulated the expression and phosphorylation levels of key proteins (e.g., p‐PI3K, p‐AKT, and p‐mTOR) within this pathway in the skeletal muscle or relevant tissues of CIF model mice (all *p*  < 0.05).

**Conclusion:**

XLK enhances cellular energy homeostasis by regulating the PI3K/AKT‐mTOR‐FoxO signaling axis, thereby alleviating CIF. These findings provide a mechanistic rationale for the clinical application of XLK against CIF.

## 1. Introduction

Chemotherapy‐induced fatigue (CIF) is a prevalent and debilitating side effect in cancer patients, referring to persistent, subjective exhaustion directly caused by chemotherapeutic agents (e.g., 5‐FU), and severely compromises quality of life and treatment adherence [1, 2]. Its pathogenesis is multifactorial, with growing evidence implicating systemic dysregulation of energy metabolism as a central contributor. Energy metabolism disorders involve disruptions in the cellular processes of energy production and utilization, manifested as impaired glycogen synthesis, lactic acid accumulation, and insufficient ATP generation [3–6]. Despite its clinical significance, effective pharmacological management of CIF remains a challenge, as existing options often provide limited symptomatic relief without addressing the underlying metabolic disturbances [7]. Therefore, elucidating the molecular pathways driving metabolic dysfunction in CIF is crucial. This knowledge will facilitate the development of targeted therapies.

Traditional Chinese medicine (TCM) offers a complementary approach with potential for multitarget intervention. From a TCM perspective, CIF aligns with the syndrome of “deficiency‐consumption” resulting from pathogenic toxin invasion and vital qi depletion [8, 9]. Herbal formulations, through their synergistic multicomponent actions, have demonstrated promise in mitigating cancer‐related fatigue (CRF) by holistically restoring balance [10–13].

The Xinlikang (XLK) capsule (National Drug Approval Number Z20080623, Guiyang Xintian Pharmaceutical Co., Ltd.) is a standardized multiherb formulation consisting of 10 medicinal components, including *Scutellaria barbata*, *Astragalus membranaceus*, *Angelica sinensis*, and *Salvia miltiorrhiza*. The formula is derived from a traditional Miao ethnic prescription in Guizhou Province and has been used clinically for nearly two decades as an adjuvant therapy to mitigate chemotherapy‐related complications [14]. Previous studies by our group have confirmed its immunomodulatory and antitumor properties [15–17]. However, its specific efficacy against CIF and its potential mechanism of action have not been scientifically elucidated.

Multiple studies have shown that the PI3K/AKT‐mTOR signaling axis is a master regulator of cellular growth, survival, and metabolism. Crucially, it plays a pivotal role in governing glucose homeostasis and energy balance [18, 19]. Glycogen storage refers to the form of glucose stored in the liver and muscles, serving as the primary energy source for physical activity. Activation of this pathway promotes glycogen synthesis, enhances cellular glucose uptake, and modulates glycolytic flux, processes that are frequently disrupted in CIF [20–22]. Consequently, targeted modulation of the PI3K/AKT‐mTOR pathway and its interconnected partner FoxO has emerged as a rational strategy for rectifying metabolic imbalances and alleviating fatigue. Notably, a preliminary network pharmacology analysis of XLK’s components predicted a significant convergence of its potential targets on this very pathway, highlighting it as a prime candidate for mechanistic investigation.

To bridge this knowledge gap, the present study employed an integrated strategy combining experimental validation and network pharmacology prediction. Using a 5‐FU‐induced murine model of CIF, we comprehensively evaluated the anti‐fatigue effects of XLK through behavioral, histological, and metabolic assays. Furthermore, we systematically investigated whether its therapeutic benefits were mediated through the modulation of the PI3K/AKT‐mTOR‐FoxO signaling axis. Our findings aim to provide a mechanistic foundation for the clinical application of XLK in the management of CIF.

## 2. Results

### 2.1. Pharmacodynamic Evaluation of XLK in CIF Mice

#### 2.1.1. Effects of XLK on Body Weight and Behavior of CIF Mice

The animal experimental design is shown in Figure 1A. Dynamic observation of the physical and mental state of the mice during the experimental period revealed that the mice in the CIF model group showed symptoms such as erect hair, slow movement, poor mood, and weight loss, and the above symptoms were alleviated in the XLK administration group. In addition to behavioral observations, we also performed extensive behavioral tests. Body weight, food and water intake, and exercise endurance (weight‐bearing swimming time, tail suspension time, and grip strength) of the model group were dramatically lower than those of the blank control group (*p*  < 0.01, Figure 1B−H); except for the food and water intake index, the medium‐ and high‐dose XLK groups could significantly reverse the above symptoms (*p* < 0.05). Compared with the model group, the food and water intake of each treatment group showed an increasing trend, but there was no significant difference (Figure 1C,F). Taken together, these results suggest that XLK is effective in alleviating the behavioral abnormalities associated with CIF.

Figure 1Effects of XLK on the behavior of CIF mice. (A) Animal experimental procedure. (B) Body weight change. (C) Food intake. (D) Weight‐bearing swimming. (E) Tail suspension. (F) Water intake. (G) Horizontal suspension. (H) Grip strength. Data were analyzed using one‐way ANOVA mean ± standard deviation (*n =* 10). ^#^ *p* < 0.05, ^##^ *p* < 0.01 vs. blank control group;  ^∗^ *p* < 0.05,  ^∗∗^ *p* < 0.01 vs. model group.

**Figure:**
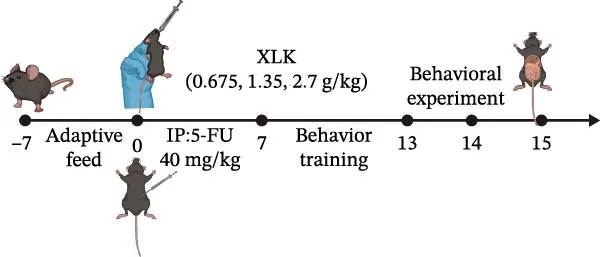
(A)

**Figure:**
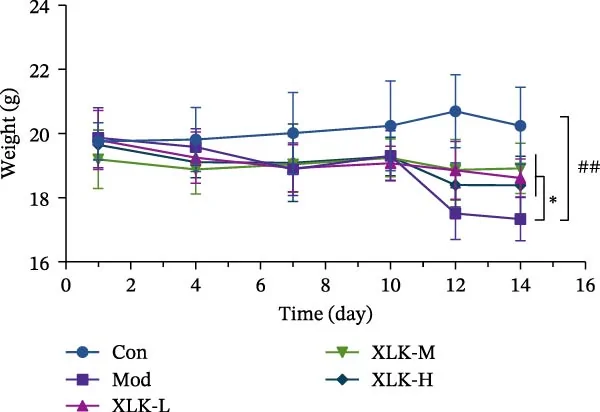
(B)

**Figure:**
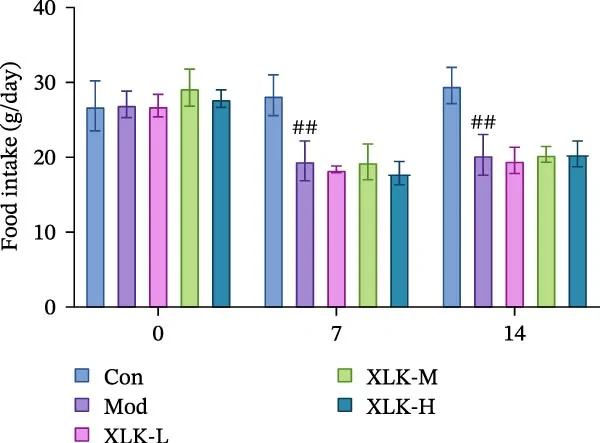
(C)

**Figure:**
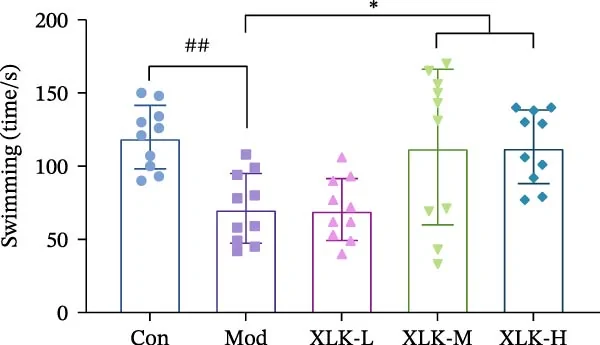
(D)

**Figure:**
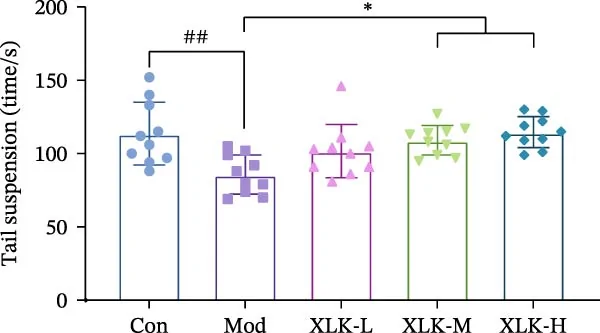
(E)

**Figure:**
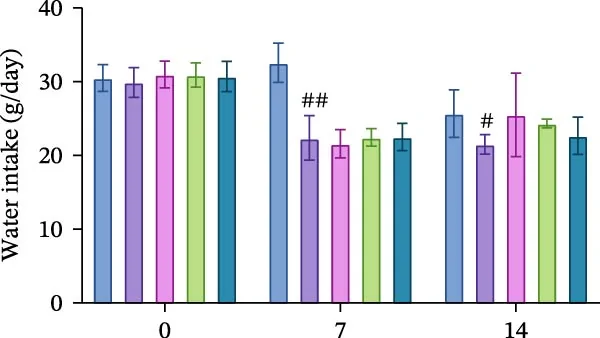
(F)

**Figure:**
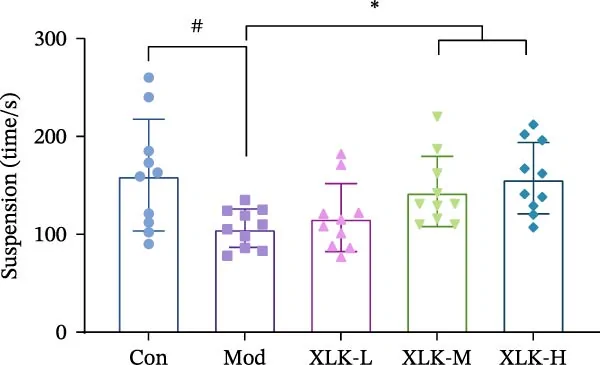
(G)

**Figure:**
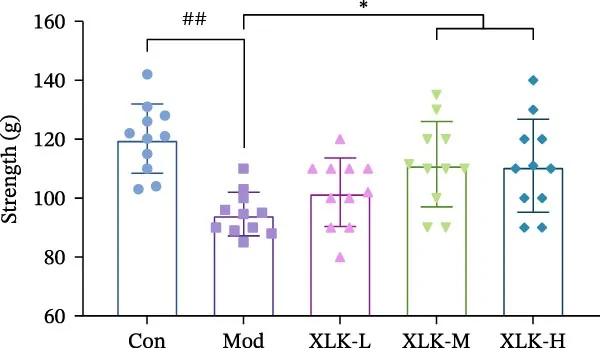
(H)

#### 2.1.2. Effect of XLK on Pathological Changes and Organ Index of Gastrocnemius Muscle in CIF Mice

Muscle fatigue causes lesions in gastrocnemius muscle tissue. HE staining of mice in each group showed that in the gastrocnemius muscle tissue of untreated control mice, the boundaries between myofibers were clear, the transverse striations were bright, and the nuclei were located at the edges; in the model group, the cells were found locally, with fragmented and lysed nuclei, increased cytoplasmic eosinophilia, and a small number of mast cells and granulocytes infiltrating the mesenchyme, with a decrease in the volume of myofibroblasts in the local area (Figure 2A). These pathological manifestations were consistent with the changes in muscle fatigue induced by CIF, indicating successful modeling. The above symptoms were alleviated after drug administration. Analysis of mouse gastrocnemius muscle fibers bundles showed that the cross‐sectional area of the gastrocnemius muscle in the model group was significantly reduced compared with that in the blank control group (*p* < 0.01), and the cross‐sectional area of the gastrocnemius muscle in the middle and high dose groups of XLK could be significantly increased compared with that in the model group (*p* < 0.05, Figure 2B). The results of organ indices showed that compared with the blank control group, all organ indices in the model group showed a decreasing trend, but there was no significant difference; compared with the model group, there was an increasing trend in the organ indices of the XLK‐treated group, among which the medium‐dose group was significant (*p* < 0.05), and there was no significant difference among the other groups (Figure 2C−E). The above results indicated that XLK could superiorly improve the gastrocnemius muscle histopathology in CIF mice.

Figure 2Effect of XLK on gastrocnemius muscle tissue of CIF mice. (A) HE staining of gastrocnemius muscle tissue. (B) Cross‐sectional area of gastrocnemius muscle. (C) Liver index. (D) Spleen index. (E) Renal index. Data were analyzed using one‐way ANOVA mean ± standard deviation (*n* = 6). ^##^ *p* < 0.01 vs. blank control group;  ^∗^ *p* < 0.05 vs. model group.

**Figure:**
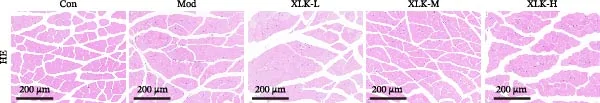
(A)

**Figure:**
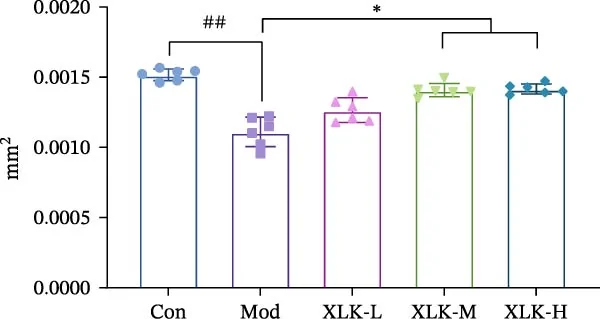
(B)

**Figure:**
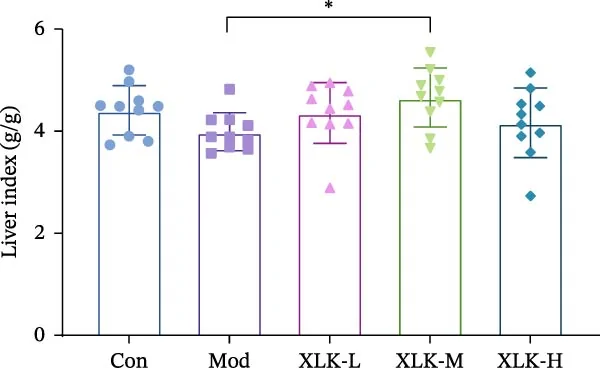
(C)

**Figure:**
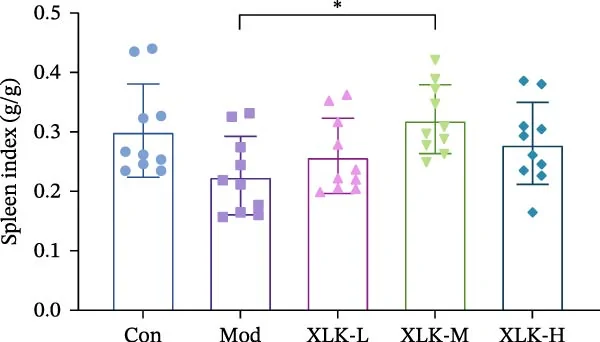
(D)

**Figure:**
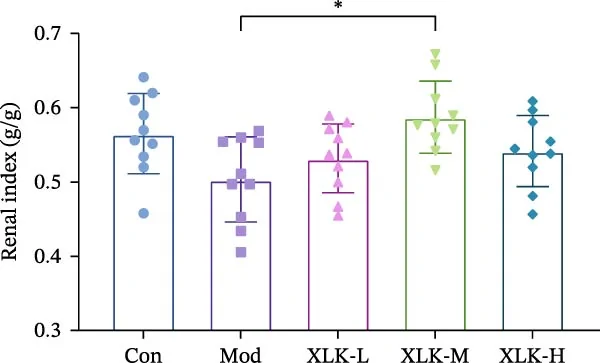
(E)

#### 2.1.3. Effect of XLK on Glycogen Storage in CIF Mice

Glycogen is the main energy source, and insufficient glycogen storage exacerbates fatigue and impairs exercise capacity. Periodic acid‐Schiff (PAS) staining of liver and gastrocnemius muscle showed that the liver glycogen and muscle glycogen contents of the model group were significantly reduced compared with the blank control group, and the medium‐ and high‐dose XLK groups could significantly increase the contents of liver glycogen and muscle glycogen (*p* < 0.05, Figure 3B,C).The ELISA results showed that compared with the blank control group, the glycogen content of the liver and gastrocnemius muscle tissues of the model group was dramatically reduced (*p* < 0.05), and compared with the blank control group, hepatic and myofibrillar glycogen contents in the model group were dramatically reduced (*p* < 0.05). The medium‐ and high‐dose XLK groups significantly elevated hepatic glycogen (*p* < 0.05), while the high‐dose XLK group further increased myofibrillar glycogen (*p* < 0.05, Figure 3D,E), which was consistent with the results of PAS staining. These results suggest that XLK can effectively improve glycogen storage in CIF.

Figure 3Effect of XLK on glycogen storage in CIF mice. (A) PAS staining of gastrocnemius and liver tissues (scale bar = 200 μm). (B) Semiquantitative PAS of liver tissues. (C) Semiquantitative PAS of gastrocnemius tissues. (D) Hepatic glycogen content (normalized to tissue weight). (E) Myogenic glycogen content (normalized to tissue weight). Data were analyzed using one‐way ANOVA mean ± standard deviation (*n* = 6). ^#^ *p* < 0.05 vs. blank control group;  ^∗^ *p* < 0.05 vs. model group.

**Figure:**
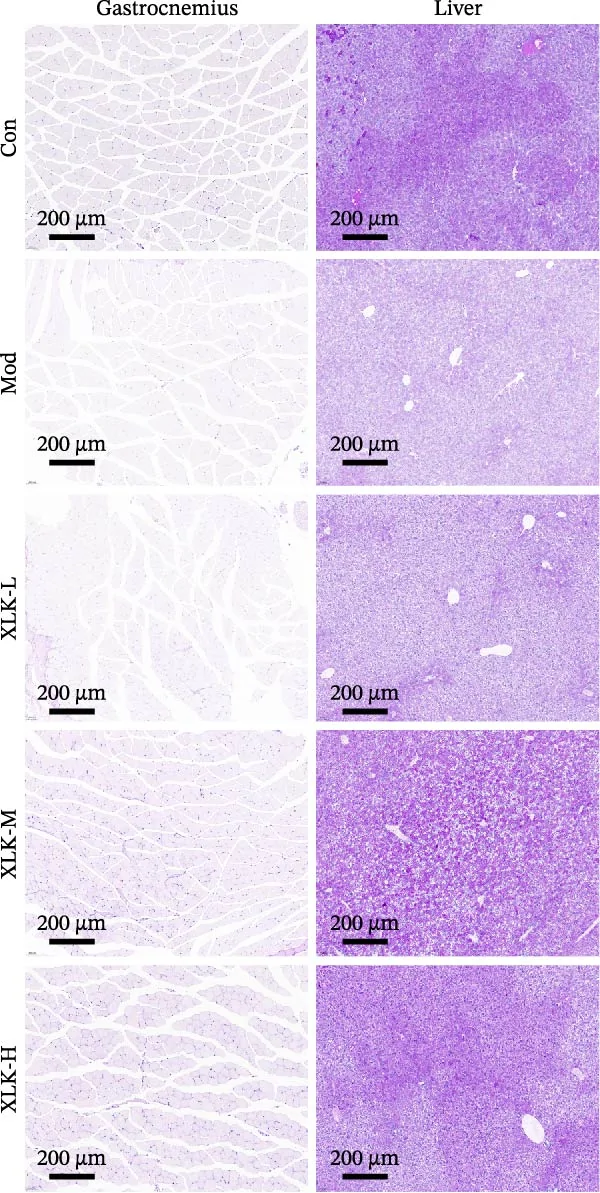
(A)

**Figure:**
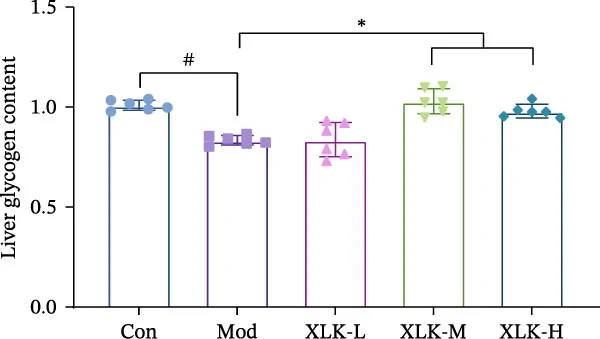
(B)

**Figure:**
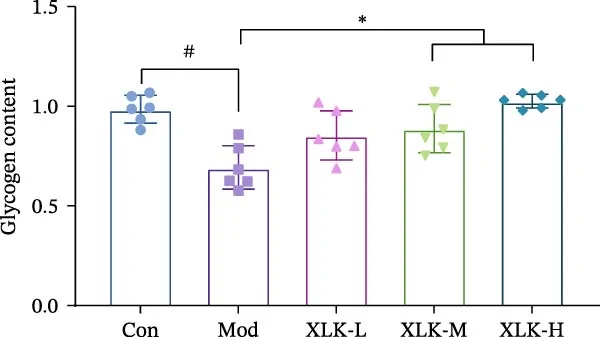
(C)

**Figure:**
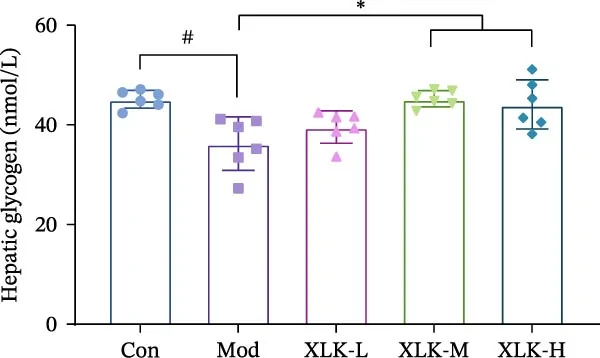
(D)

**Figure:**
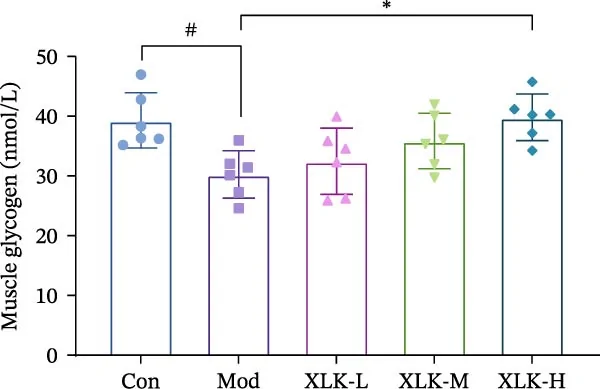
(E)

#### 2.1.4. Effects of XLK on Lactic Acid and Energy Metabolism in CIF Mice

Lactate accumulation was closely associated with increased fatigue: the ELISA results showed that the blood lactate content in the model group was significantly increased compared with the blank control group (*p* < 0.05), and the area under the blood lactate curve was profoundly decreased in each administration group compared with the model group (*p* < 0.01, Figure 4B). Overall, at different time points, each administration group could significantly improve the body’s ability to metabolize lactate and keep lactate in a relatively balanced state compared with the model group (Figure 4A). During exercise, proper control of lactate production and removal is important for maintaining ATP stability and improving exercise performance. Similarly, the increased reserve of glycogen as a direct source of energy for muscle activity (Figure 3) can prolong the exercise endurance of mice by improving the efficiency of ATP production (Figure 4C) and thus prolong the exercise endurance of mice (Figure 1D,E). It was found that the ATP content in the model group was significantly reduced compared with the blank control group (*p* < 0.05); compared with the model group, the medium and high dose XLK groups could significantly increase the ATP content of the organism (*p* < 0.05) (Figure 4C). Concurrently, the medium‐dose XLK effectively increased hemoglobin (HGB) and erythropoietin (EPO) levels, while the high dose of XLK has also been demonstrated to effectively increase the content of HGB in the body (*p* < 0.05, Figure 4E,F). This, in turn, enhances the body’s capacity for oxygen transportation, thereby improving energy production. Overall, XLK could effectively improve the abnormal lactate metabolism in CIF mice.

Figure 4Effect of XLK on lactate and energy metabolism in CIF mice. (A) Change in lactate content. (B) Lactate content. (C) Liver ATP content (normalized to tissue weight). (D) CRP content. (E) HGB content. (F) EPO content. Data were analyzed using one‐way ANOVA mean ± standard deviation (*n* = 6). ^#^ *p* < 0.05, ^##^ *p* < 0.01 vs. blank control group;  ^∗^ *p* < 0.05,  ^∗∗^ *p* < 0.01 vs. model group.

**Figure:**
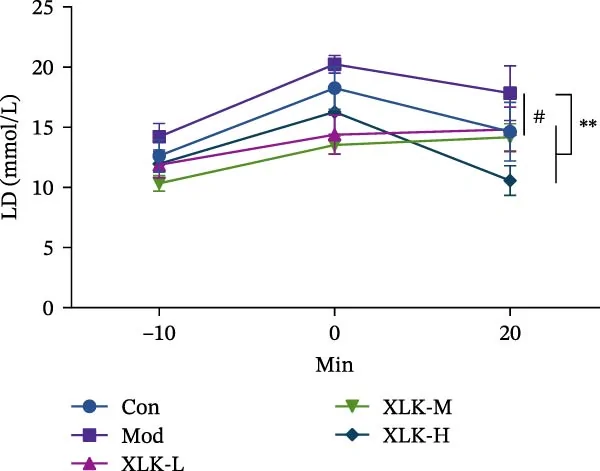
(A)

**Figure:**
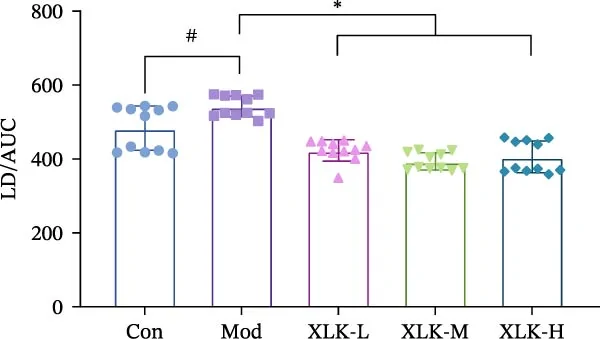
(B)

**Figure:**
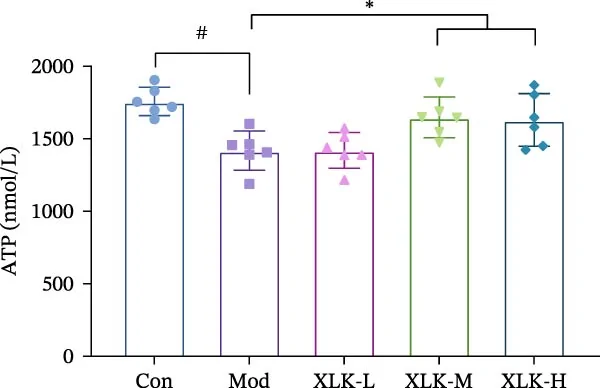
(C)

**Figure:**
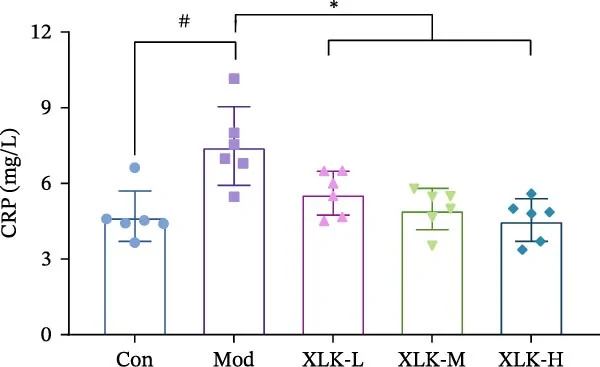
(D)

**Figure:**
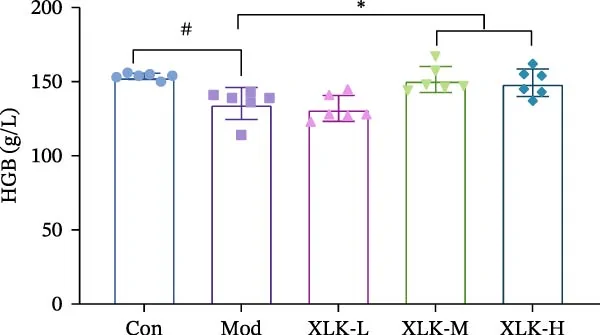
(E)

**Figure:**
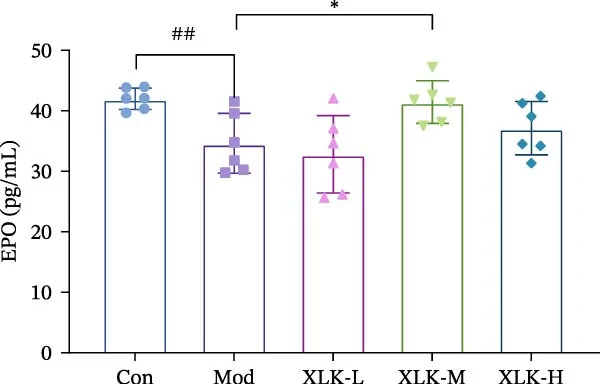
(F)

### 2.2. Network Pharmacology‐Based Prediction of the Mechanisms Involved in the Amelioration of CIF by XLK

#### 2.2.1. Collection of Potential Targets for XLK to Improve CIF

The experimental results presented above have confirmed at the whole animal level that XLK has the capacity to alleviate CIF. In consideration of the complexity of the components of TCM and the holistic nature of its action, it may be hypothesized that XLK could improve CIF through multicomponent, multitarget, and multipathway mechanisms. The results of XLK were analyzed by the TCMSP and Swiss Target Prediction databases with pharmacokinetics. A total of 781 component‐related targets were retrieved from the TCMSP and Swiss Target Prediction databases (screened by pharmacokinetic properties and druglikeness), and target names were standardized using the UniProt database. Utilizing the search term “Chemotherapy‐induced Fatigue” (CIF), a comprehensive set of CIF‐related targets was obtained from the DisGeNET, OMIM, and GeneCards databases. Following the elimination of duplicates, a total of 3913 disease targets were identified. The compositional and disease targets were imported into the online Venny tool in order to create a Venn diagram, and 411 intersecting targets were eliminated (Figure 5A). Concurrently, the XLK “component‐target” network was constructed (Figure 5B), and protein–protein interaction (PPI) analysis revealed that XLK exerted its influence on 96 core targets, including AKT1, ESR1, EGFR, INSR, CASP3, MTOR, and others (Figure 5C). Pursuant to the topological analysis, the top 30 key components, including 4‐hydroxycoumarin, chrysin, and baicalein, were identified (Table S2).

Figure 5Network pharmacology predicts relevant targets for XLK to improve CIF. (A) Screening of intersection targets of XLK to improve CIF. (B) Screening of component‐core targets of XLK to improve CIF. (C) Predicting core targets of XLK to improve CIF.

**Figure:**
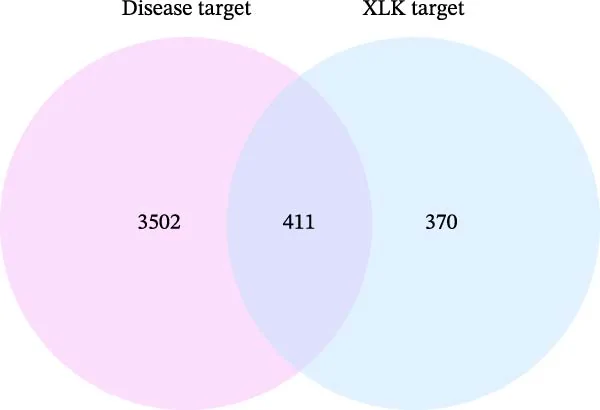
(A)

**Figure:**
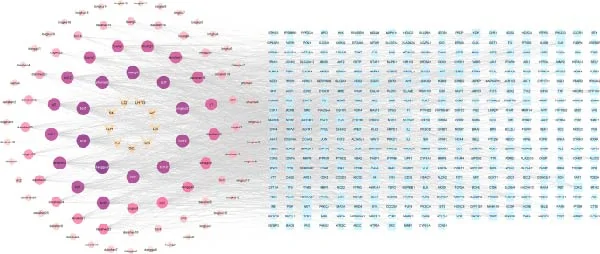
(B)

**Figure:**
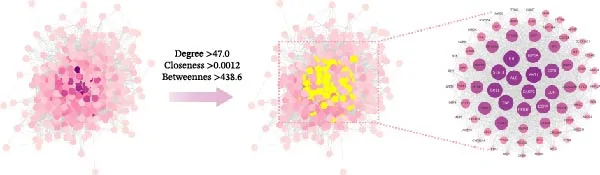
(C)

#### 2.2.2. Gene Ontology (GO) Functional Annotation and Kyoto Encyclopedia of Genes and Genomes (KEGG) Enrichment Analysis of XLK to Improve CIF

We performed GO and KEGG enrichment analyses on 96 core targets to explore the potential mechanism by which XLK ameliorates CIF (Figure 6A). In addition, KEGG pathway enrichment analysis showed that 20 pathways were most significantly enriched, among which the PI3K/AKT pathway, mTOR pathway and FoxO pathway were more significant (Figure 6B). A review of the literature revealed that the PI3K/Akt pathway regulates cell metabolism, proliferation, etc., and mTOR is an energy sensor involved in aerobic glycolysis and transcription of tumor growth genes, etc. [23]. Based on the core targets, the results of the analysis of the key pharmacodynamic components of XLK (Table S3) showed that 7,2′‐dihydroxy‐3′, 4′‐dimethoxyisoflavanes could regulate MTOR, AKT1, EGFR, and CASP3; quercetin and isomauritiflorin target of AKT1, INSR, and EGFR; tanshinone IIB may regulate EGFR, INSR, and other core targets; hemimelanin K may regulate MTOR, EGFR, and ESR1 and other core targets; baicalein and apigenin can regulate AKT1, EGFR, and ESR1 and other core targets; and quercetin, 6‐hydroxycoumarin, 4‐hydroxycoumarin, 8‐hydroxymuramicin, arbutin, and other components can regulate AKT1, ESR1, EGFR, INSR, and other core targets. Targets, suggesting that these compounds play an important role in improving CIF in mice. Follow‐up experiments verified the molecular mechanism of CIF treatment based on XLK compounds in the PI3K/AKT‐mTOR‐FoxO signaling pathway (Figure 6C).

Figure 6Network pharmacology predicts the related pathways of XLK improving CIF. (A) GO analysis of CIF improvement by XLK. (B) KEGG analysis of CIF improvement by XLK. (C) Modulation of PI3K/AKT‐mTOR‐FoxO signaling pathway by XLK active ingredients.

**Figure:**
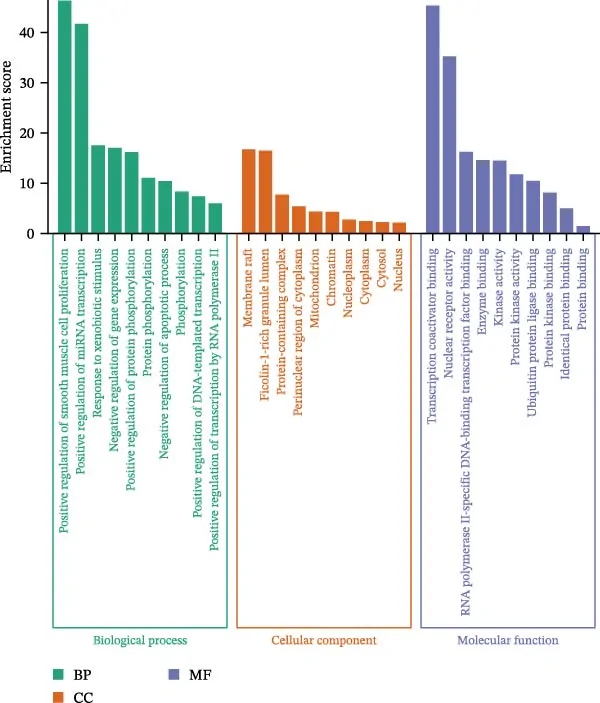
(A)

**Figure:**
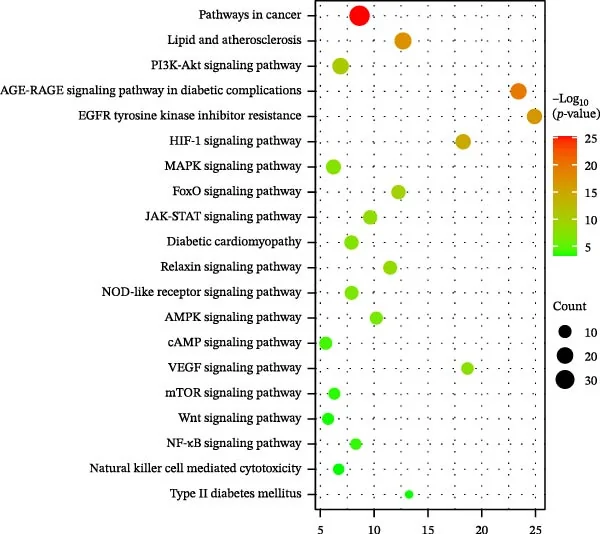
(B)

**Figure:**
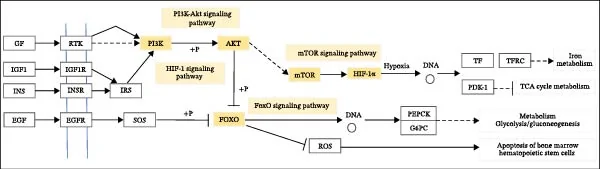
(C)

#### 2.2.3. XLK Regulates Organismal Energy Metabolism Through the PI3K/Akt‐mTOR‐FoxO Pathway

In order to ascertain whether XLK enhanced CIF in mice by modulating PI3K/AKT‐mTOR‐FoxO, a western blot analysis was conducted. As demonstrated in Figure 7, the protein expression levels of PI3K, p‐AKT, mTOR, and S6K in the tissues of the model group were significantly increased (*p* < 0.05). In addition, the protein expression level of FoxO was significantly decreased (*p* < 0.05, Figure 7G). However, no evident difference in AKT protein expression was observed between the groups (Figure 7F). In comparison with the model group, both the medium‐ and high‐dose groups demonstrated a significant reduction in the protein expression of PI3K, p‐AKT, mTOR, and S6K in gastrocnemius muscle tissues (*p* < 0.01, Figure 7B–E). Furthermore, the low‐dose group exhibited a significant reduction in the protein expression levels of p‐AKT and PI3K in tissues (*p* < 0.05, Figure 7B,C). In contrast, the medium‐ and high‐dose groups significantly elevated the protein expression levels of FoxO in tissues (*p* < 0.01, Figure 7G). The findings suggested that XLK could enhance CIF via the PI3K/Akt‐mTOR‐FoxO signaling pathway.

Figure 7Effect of XLK on protein expression in gastrocnemius muscle tissues of CIF mice. (A) WB bands. (B) mTOR relative expression. (C) PI3K relative expression. (D) FoxO relative expression. (E) (p70)S6K relative expression. (F) p‐AKT relative expression. Data were analyzed using one‐way ANOVA mean ± standard deviation (*n = 6*). ^#^ *p* < 0.05, ^##^ *p* < 0.01 vs. blank control group;  ^∗^ *p* < 0.05,  ^∗∗^ *p* < 0.01 vs. model group.

**Figure:**
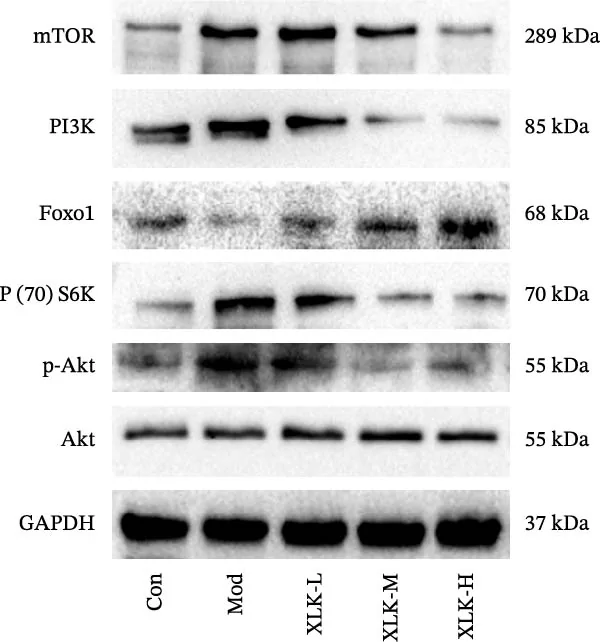
(A)

**Figure:**
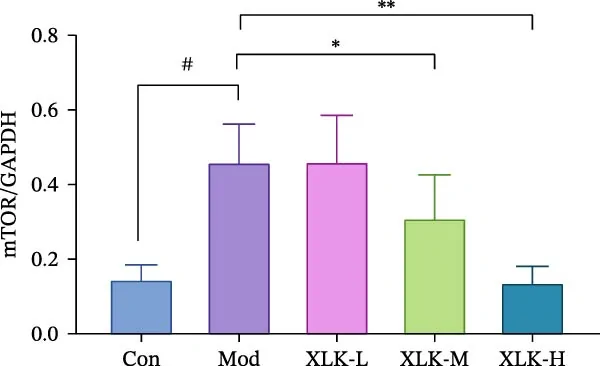
(B)

**Figure:**
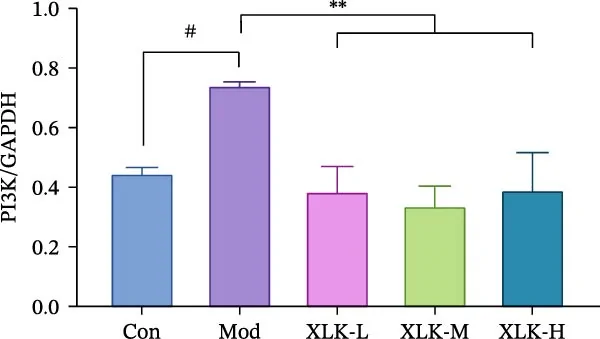
(C)

**Figure:**
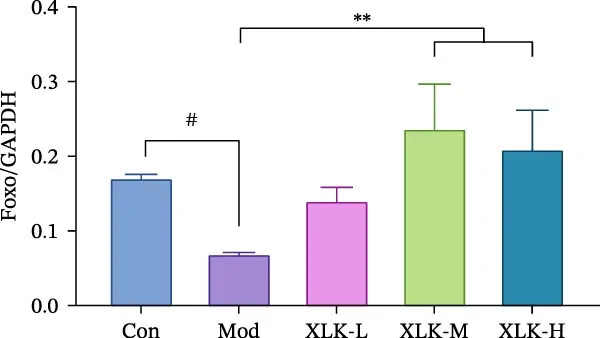
(D)

**Figure:**
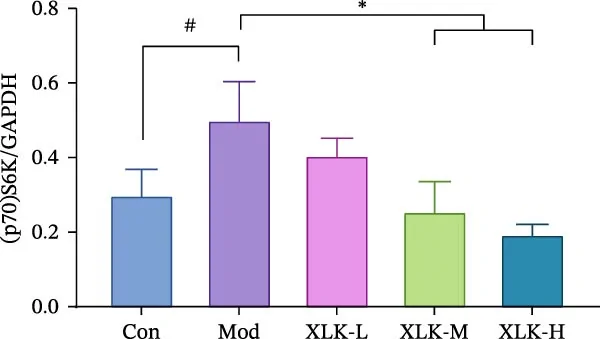
(E)

**Figure:**
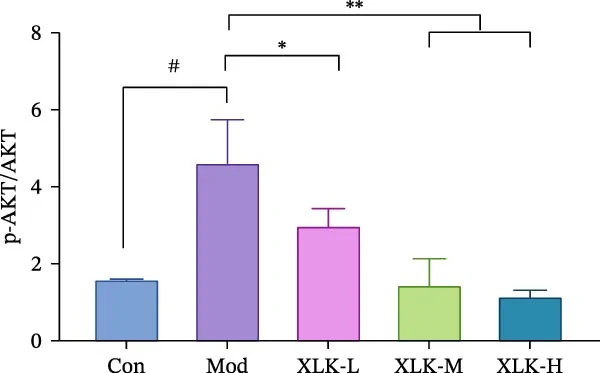
(F)

#### 2.2.4. Discussion

Cancer remains a major focus in medical research and therapeutic development worldwide. While chemotherapy serves as the cornerstone of oncological treatment, it induces CIF in 80%–90% of patients during therapeutic regimens [24]. Clinical studies have demonstrated XLK efficacy in alleviating fatigue symptoms among cancer chemotherapy patients [14, 15], though the underlying mechanisms remain unclear. Through integrated animal experiments and network pharmacology approaches, this study elucidated XLK therapeutic effects on CIF. XLK significantly enhanced exercise endurance in CIF murine models while mitigating chemotherapy‐associated weight loss and muscle atrophy. Notably, we identified its potential mechanistic basis for the first time, providing critical pharmacological evidence to inform clinical practice and facilitate drug development in this field.

Glycogen serves as the primary energy substrate for muscular activity, with hepatic glycogen mobilization occurring upon depletion of myofiber glycogen storage. Significant reductions in both muscular and hepatic glycogen storage predispose organisms to metabolic dysregulation [25]. The metabolite accumulation hypothesis posits that high‐intensity exercise demands rapid ATP regeneration, driving anaerobic glycolysis to produce excess lactate. This pathophysiological process induces cellular damage through acidotoxicity and exacerbates fatigue [26, 27]. Mechanistically, XLK improves CIF via a tripartite metabolic modulation: (1) increasing hepatic glycogen, (2) increasing myofibrillar glycogen, and (3) increasing ATP production. These results demonstrate XLK’s ability to optimize bioenergetic homeostasis and thereby improve exercise tolerance in the pathophysiology of CIF.

The PI3K/AKT pathway, a central regulator of energy metabolism, maintains dynamic equilibrium between glycolysis and oxidative phosphorylation through the phosphorylation of mTOR and FoxO by its downstream effector Akt [19, 28, 29]. It is important to note that mTOR has been shown to stimulate the transcriptional activation of genes associated with aerobic glycolysis and tumor progression, including HIF‐1 α and NF‐κB [30, 31]. As a pivotal downstream target of the mTOR pathway, S6K plays a critical role in regulating cellular growth processes [32]. Conversely, elevated FoxO expression has been shown to suppress glycolytic activity while enhancing oxidative phosphorylation‐mediated glucose metabolism to generate substantial ATP [33, 34]. Moreover, FoxO has been shown to possess regulatory capacity in bone marrow hematopoietic stem cell apoptosis and renewal. Integrative network pharmacology analysis elucidated the anti‐fatigue mechanism of XLK against CIF. Through the construction of herb‐component‐target networks and core target‐component networks, it was found that baicalein, apigenin, quercetin, tanshinone IIB, dihydrocurcumin, and so on could be the key active ingredients of XLK. These phytochemicals showed synergistic regulation of key targets, including AKT and mTOR, by modulating PI3K/AKT‐mTOR‐FoxO signaling pathways.

This mechanism is highly consistent with the previously reported anti‐fatigue effects of quercetin and ginsenosides [35, 36], and it has been shown that *Panax ginseng* polysaccharides and *Lycium barbarum* polysaccharides can alleviate fatigue by increasing glycogen and regulating metabolism [35, 37, 38]. However, the unique advantage of XLK lies in its multicomponent synergistic regulation of multiple targets such as AKT and mTOR, which may be more applicable to the complex pathological environment of CIF.

The novelty of this study lies in two aspects [1]: For the first time, we systematically elucidated the molecular mechanism of XLK against CIF, confirming that it regulates the PI3K/AKT‐mTOR‐FoxO pathway to improve energy metabolism homeostasis. This finding fills a critical gap in prior research regarding the anti‐fatigue effects of XLK [2]. We integrated network pharmacology and in vivo experiments to identify key active components (baicalein, apigenin, quercetin) and core targets (AKT, mTOR, and FoxO) of XLK, revealing its multi‐component synergistic regulatory mode, which is more suitable for the complex pathological environment of CIF (Table S4). XLK is an approved clinical adjuvant drug for radiotherapy and chemotherapy, with a proven safety profile (previous studies have shown no obvious toxicity in animal models and clinical use). The 5‐FU‐induced CIF model used in this study simulates the fatigue state of clinical chemotherapy patients, and the key mechanism (PI3K/AKT‐mTOR‐FoxO pathway regulation) is conserved between mice and humans. In subsequent experiments, the therapeutic efficacy of XLK in alleviating CRF induced by combination chemotherapy was concurrently validated across multiple tumor models, including colorectal and lung cancers. Thus, XLK has good clinical translatability.

The present study is not without its limitations. Several limitations should be considered when translating the findings to clinical practice [1]: The animal model cannot fully replicate the complexity of clinical CIF (e.g., patients may have comorbidities such as anemia, inflammation, or malnutrition, which affect fatigue) [2]. The study used a single chemotherapy drug (5‐FU); XLK’s efficacy in other chemotherapy regimens (e.g., paclitaxel, cisplatin) remains unclear. Future studies should address these limitations through multicenter clinical trials, dose‐escalation studies, and subgroup analyses of different tumor types/chemotherapy regimens.

#### 2.2.5. Conclusion

**Figure 8:**
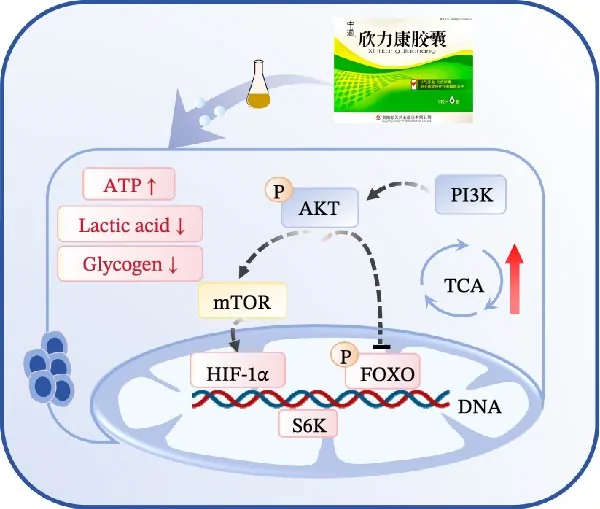
XLK alleviates CIF via the PI3K/AKT‐mTOR‐FoxO pathway.

This study, which combines network pharmacology and holistic pharmacodynamic analysis, demonstrates that XLK effectively reduces lactate accumulation, restores cellular energy homeostasis, and ameliorate chemotherapy‐induced weight loss and fatigue behaviors via the PI3K/AKT‐mTOR‐FoxO signaling pathway, ultimately alleviating CIF (Figure 8).

## 3. Materials and Methods

### 3.1. Instruments and Reagents

Micro CL21R high‐speed freezing centrifuge (Thermo Fisher Scientific, China); 30002 electronic balance (Hangzhou Youheng Weighing Equipment Co., Ltd.); Synergy H1 multifunctional microplate tester (Berten Instruments, Inc., USA); gripping force tester (Jinan Yiyan Science and Technology Development Co., Ltd.); TS‐200 Suspension Tester (Anhui Zhenghua Biological Instrument Co., Ltd.); 5‐Fluorouracil (5‐FU) (Sangong Bioengineering (Shanghai) Co., Ltd., Batch No. J913BA0017); XLK capsule (National Drug Approval Number Z20080623, Guiyang Xintian Pharmaceutical Co., Ltd., Batch Number 230601); LD‐Lactic Acid Kit (Nanjing Jiancheng Bioengineering Institute, Batch Number 20240417); Glycogen, ATP ELISA kit (Shanghai Yuanjang Biotechnology Center, Batch No.: 202406); PI3 Kinase p110 Beta Polyclonal antibody (Batch Number: 20584–1‐AP), Phospho‐AKT (Ser473) Recombinant antibody (Batch Number: 28731–1‐AP), p70(S6K) Polyclonal antibody (Batch Number: 14485–1‐AP) were purchased from ProteinTech (Wuhan, China); Akt (pan) (C67E7) Rabbit mAb (Batch No: 4691 T), mTOR (7C10) Rabbit mAb (Batch Number: 2983 T), FoxO1 (C29H4) Rabbit mAb (Batch Number: 2880 T) was purchased from Cell Signaling Technology (Massachusetts, USA).

### 3.2. Experimental Animals

C57BL/6 female mice (*n =* 150; age: 6 weeks; body weight: 18–22 g) were obtained from Jiangsu Qinglongshan Biotechnology Co., Ltd. (Animal Production License: SCXK(Su)2024−0001). Animals were housed in specific pathogen‐free (SPF) facilities under controlled environmental conditions (temperature: 22–24°C; relative humidity: 45%–80%) with a 12/12 h light/dark cycle. Following a 7‐day acclimatization period, all experimental procedures were conducted in strict compliance with the ARRIVE guidelines and approved by the Animal Ethics Committee of Nanjing University of Chinese Medicine (Approval Number 202404A038).

### 3.3. Animal Experiments

C57BL/6 female mice (SPF grade) were randomly assigned to five experimental cohorts (*n =* 30 per group) using a randomized block design [1]: blank control [2], CIF model [3], XLK low‐dose (0.675 g/kg) [4], XLK medium‐dose (1.35 g/kg; this dose is the clinically equivalent dose of XLK, instructions for use are detailed in the Supplementary Data), and [5] XLK high‐dose (2.7 g/kg). The CIF model was induced via daily intraperitoneal injection of 5‐FU (40 mg/kg) for seven consecutive days in all groups except the blank control. Concurrently, XLK‐treated groups received oral gavage of XLK at the respective doses for a period of 14 days, while control and model groups received equivalent volumes of sterile saline. Throughout the experimental timeline, behavioral and physiological parameters were monitored on a daily basis. These parameters included locomotor activity, integumentary integrity and nutritional intake (food/water consumption).

### 3.4. Indicators of Drug Efficacy

#### 3.4.1. Grip Strength Test

The measurement of forelimb grip strength was conducted by means of a digital dynamometer, the purpose of which was to assess neuromuscular coordination and muscle function. On experimental Day 13, 10 mice per cohort underwent standardized grip strength testing. The subjects were positioned on the testing platform with their forepaws grasping the traction bar. In accordance with the ISO 15194 calibration protocols, the experimenter maintained the animal’s hindquarters while gradually applying posterior traction until grip release occurred. The maximum peak force (in Newtons) was recorded across five consecutive trials, with the highest value retained for statistical analysis.

#### 3.4.2. Tail Suspension Test

Following a 6 h recovery period after grip strength assessment, 10 mice per cohort underwent tail suspension testing. Using the TS‐200 Behavioral Analysis System, the distal 2 cm of the tail was attached to the suspension bar with medical grade tape, maintaining a 5 cm vertical distance between the animal’s cephalic region and the base of the apparatus. Immobility latency was quantified as the duration of active escape behavior (including body torsion and limb motility) that persisted within a standardized 6‐min observation window.

#### 3.4.3. Horizontal Suspension Test

The horizontal suspension test was used to assess neuromuscular parameters, including grip strength, balance, endurance, forelimb strength and motor coordination. On experimental Day 14, ten mice per cohort underwent standardized testing using a suspended horizontal grid apparatus positioned 30 cm above the substrate. Subjects were positioned to grasp the grid with their forelimbs, with three consecutive trials performed at 5 min intervals. Maximum suspension latency was recorded as the primary endpoint, defined as the longest duration of sustained forelimb engagement observed in triplicate measurements.

#### 3.4.4. Weight‐Bearing Swimming

The improvement in exercise endurance served as the primary indicator of improved fatigue resistance. On experimental Day 14, following a 6 h recovery period after the grip strength test, 10 mice per cohort underwent a weighted swimming assessment. A lead weight equivalent to 10% of individual body mass was attached to the base of the tail using a nonrestrictive attachment. Subjects were then placed in a temperature‐controlled water maze (50 × 50 × 40 cm; water depth: 30 cm; 25 ± 1°C). Swimming latency was quantified as the time from initiation of immersion to the exhaustion endpoint, characterized by loss of purposeful locomotion and failure to maintain buoyancy.

#### 3.4.5. Blood Lactate Measurement

Retro‐orbital blood samples were taken at three defined intervals: (1) 2 h after the last administration (baseline), (2) immediately after 10 min of forced swimming in a thermoregulated water chamber (30 ± 0.5°C), and (3) after 20 min of recovery. Venous blood (50 μL per sample) was collected using heparinized capillary tubes. Serum lactate concentrations were quantified at baseline (*T*_0_), immediately after exercise (*T*_10_) and during recovery (*T*_20_) using a commercial lactate assay kit. The lactate accumulation index was calculated using trapezoidal integration as follows:

AUC=12LT0+LT10×Δt1+12LT10+LT20×Δt2,

where Δ_*t*1_ = 10Δ_*t*1_ = 10 min (exercise duration) and Δ_*t*2_ = 20 min (recovery period).

#### 3.4.6. PAS Staining for Liver and Muscle Glycogen Content

After terminal blood sampling, mice were euthanised by cervical dislocation and surface disinfected with 75% ethanol for 5 min. Liver and gastrocnemius muscle samples were dissected, rinsed in ice‐cold phosphate‐buffered saline (PBS, pH 7.4), blotted on sterile filter paper and processed sequentially through: (1) fixation in Carnoy’s solution (ethanol:chloroform:acetic acid = 6:3:1) for 24 h, (2) a graded ethanol series (70%−100%), (3) xylene clearing, and (4) paraffin embedding. Tissue sections (5 μm thickness) were deparaffinized, rehydrated, and subjected to PAS staining. Glycogen deposition was quantified by semiquantitative morphometric analysis using ImageJ software, with PAS‐positive areas expressed as a percentage per field of view.

#### 3.4.7. HE Staining of Histopathological Changes in Mouse Gastrocnemius Muscle

The gastrocnemius muscle tissues of mice in each group were harvested, fixed with 4% paraformaldehyde solution, dehydrated, paraffin‐embedded, sectioned, dewaxed, stained with hematoxylin–eosin (H&E), dehydrated, and mounted, and the images were captured and analyzed under a microscope.

#### 3.4.8. Organ Index

After terminal blood sampling, mice were euthanized by cervical dislocation and surface disinfected with 75% ethanol for 5 min. The spleen, kidneys and liver were excised under aseptic surgical conditions, and adherent muscle fascia and connective tissue were carefully dissected using microsurgical instruments. Residual blood on organ surfaces was absorbed with sterile filter paper. Organ masses were measured gravimetrically. Relative organ mass indices were calculated as follows: Equivalent formulae were applied to kidney and liver indices.

Spleen index=Spleen mass gBody mass g×100%.

#### 3.4.9. Determination of Liver and Muscle Glycogen Content

Following terminal blood sampling, the mice were euthanised by cervical dislocation and their surfaces were disinfected with 75% ethanol for 5 min. The liver and gastrocnemius muscle tissues were then dissected and rinsed in ice‐cold 0.9% saline solution (pH 7.4), before being blotted on sterile filter paper. 50 mg of tissue was homogenized, and the supernatant was collected after centrifugation. Hepatic and myofibrillar glycogen contents were quantified using a commercial assay kit according to the manufacturer’s instructions.

#### 3.4.10. Detection of ATP Levels in Liver Tissue

Hepatic ATP quantification was performed as follows: Liver tissue samples (100 mg) were homogenized in ice‐cold lysis buffer using a mechanical tissue disruptor. Homogenates were centrifuged at 12,000 × *g* for 5 min at 4°C and supernatants were collected for analysis. In 96‐well black microtiter plates, 100 μL of ATP detection reagent was aliquoted per well and equilibrated for 5 min at room temperature (25 ± 1°C). Subsequently, 20 μL tissue lysate or ATP standard was added and immediately vortex mixed (10 s). Chemiluminescence signals (RLU) were quantified using a multimode microplate reader.

### 3.5. Network Pharmacology to Predict the Mechanism of XLK to Improve CIF

#### 3.5.1. Collection of Potential Targets for XLK to Improve CIF

The identification of the components of XLK capsule was completed previously (Table S1). The SMILES structures of XLK components obtained from the preidentification were retrieved from the Pubchem database (https://pubchem.ncbi.nlm.nih.gov/) and the PharmMapper database (https://www.lilab-ecust.cn/pharmmapper/), and were introduced into the Swiss Target Prediction DisGeNET database (http://www.swisstargetprediction.ch/), PharmMapper DisGeNET database (PharmMapper), Similarity ensemble approach (SEA) DisGeNET database (SEA Search Server) to predict potential targets of XLK. Then, from GeneCards database (https://www.genecards.org/), OMIM database (http://omim.org), PharmGKB database (https://www.pharmgkb.org/), DisGeNET database (DISGENET: The most complete genomics platform ‐ Home), and DrugBank database (http://go.drugbank.com) to collect CIF‐related targets. To elucidate the interaction of XLK targets with CIF targets, we mapped Venn diagrams and screened key active ingredients.

#### 3.5.2. PPI Network Building and Key Target Screening

After constructing the PPI network using the STRING database (confidence score >0.7), we used Cytoscape 3.9.1 software to perform topological analysis. Core targets were screened based on three criteria: degree valu ≥ 2 × median (median degree = 18 and threshold = 36), betweenness centrality ≥0.1, and closeness centrality ≥0.1. (http://www.string-db.org).

#### 3.5.3. GO and KEGG Enrichment Analysis

All target proteins regulated by XLK were subjected to GO and KEGG enrichment analysis and visualized as bar graphs and bubble plots.

#### 3.5.4. Construction of an “Active Ingredient‐Target‐Pathway” Interaction Network

The top 20 pathways enriched in compounds, overlapping targets and KEGG were selected, and the network diagram of “compound ‐ overlapping target ‐ pathway” was constructed using Cytoscape 3.9.1 software, and the top 30 key compounds were screened.

#### 3.5.5. Western Blotting

Total muscle protein was extracted, protein concentration was measured by BCA protein quantification method and loading buffer was diluted for use. Separating and stacking gels were prepared per the manufacturer’s instructions. Proteins were separated by SDS‐PAGE and transferred to PVDF membranes via semi‐dry transfer. Membranes were blocked with 0.5% skimmed milk for 1 h at room temperature, then incubated with primary antibodies (mTOR, PI3K, FoxO1, S6K, p‐AKT, and AKT) at 1:1000 dilution overnight at 4°C. After three washes with TBST, membranes were incubated with secondary antibodies for 1 h at room temperature, followed by three additional TBST washes. The gray value of the protein bands was detected using a chemiluminescence imaging system.

#### 3.5.6. Statistical Analysis

SPSS 20.0 and GraphPad Prism 9 software were used for data processing and statistical presentations, and statistical data were expressed as mean ± standard deviation (*x ± s*). Comparisons between two groups of data were made using the *t-test*, and comparisons between multiple groups of data were made using one‐way ANOVA, with *p*  < 0.05 indicating that the differences were statistically significant.

## Author Contributions

**Suzhou Huang:** conceptualization, data curation, formal analysis, project administration, software, supervision, validation, investigation, writing – original draft, writing – review and editing. **Yiheng Zhang:** investigation, methodology, data curation, writing – original draft, writing – review and editing. **Tianle Ma:** validation, investigation, writing – original draft, writing – review and editing. **Lu Zhang:** conceptualization, formal analysis, writing – original draft, writing – review and editing. **Xingxing Lu:** Investigation, writing–original draft, writing – review and editing. **Huihua Fang:** writing – review and editing, writing – original draft, writing – review and editing. **Gaohong Lv:** visualization, conceptualization, writing – original draft, writing – review and editing. **Zhipeng Chen:** supervision, conceptualization, writing – original draft, review and editing. **Xin Liu, Lina Zhang, and Li Wu**: funding acquisition, project administration, resources, supervision, visualization, writing – original draft, writing – review and editing.

## Funding

The present study received financial support from the following sources: the 2024 Jiangsu Pharmaceutical Association ‘Medicine’ Research New Sound Pharmaceutical Research Project (Grant 202495083) and the school‐level project of Nanjing University of Chinese Medicine (Grant 012009240034).

## Conflicts of Interest

The authors declare no conflicts of interest.

## Supporting Information

Additional supporting information can be found online in the Supporting Information section.

## Supporting information


**Supporting Information** The Supporting Information delineate the methodological strategy employed for the component analysis of Xinlikang capsule, identify the characterized chemical constituents, and provide supporting raw data related to the network pharmacology. Table S1 XLK prototype component identification Table S2 Top 30 key components of XLK for CIF alleviation (Top 30) Table S3 Regulation of Core Targets by XLK Active Components.

## Data Availability

All relevant data is contained within the article: The original contributions presented in the study are included in the article/supporting information, further inquiries can be directed to the corresponding authors.
