# Supplementary material for: Xinlikang Capsule Alleviates Chemotherapy‐Induced Fatigue by Inhibiting the PI3K/AKT‐mTOR‐FoxO Pathway
Source: Anal Cell Pathol (Amst). 2026 Feb 19;2026:8879160. doi: 10.1155/ancp/8879160 (PMC13580801; doi:10.1155/ancp/8879160)
Supplement: Supplementary file 1 [file ANCP-2026-8879160-s001.docx]

Network-based pharmacology to explore the improvement of chemotherapy-induced fatigue by Xinlikang capsule through PI3K/AKT-mTOR-FoxO pathway

**Materials and methods**

AB SCIEX Triple TOF® 5600+ High Resolution Mass Spectrometer (AB Sciex, USA); Shimadzu 30AD Ultra High Performance Liquid Chromatograph (Shimadzu, Japan); BT125D Analytical Balance (Sartorius Scientific Instruments GmbH, Germany); Acetonitrile, methanol and formic acid were mass spectrometry grade, and water was ultrapure water; XLK capsules were supplied by Shanghai Haitian Pharmaceutical Technology Development Co Ltd (NMPA approval number Z20080623).

Preparation of test solution: A precise quantity of 1 g of XLK capsule was weighed and added to 10 mL of methanol. The mixture was then subjected to ultrasonication (power 250 W, frequency 40 kHz) in order to facilitate dissolution. Subsequent to this, the solution was filtered through a 0.22 μm filter membrane. This process resulted in the attainment of the test solution, which was then subjected to further testing. The chromatographic conditions are as follows: The Agilent C18 reversed-phase column (2.1 mm×100 mm, 1.8 µm) was eluted with a mobile phase consisting of 0.1% formic acid aqueous solution (A) and acetonitrile (B). The elution gradient was as follows: 0-25 min, 5-95% B; 25-27 min, 95-95% B; 27-29 min, 95-5% B; 29-31 min, 5-5% B. The mass spectrometric conditions encompassed electrospray ionisation (ESI) in both positive and negative ion mode scanning. The spray voltage was set at 5.5 kV (ESI+), and -4.5 kV (ESI-). The scanning range was configured from m/z 100 to 1500. The ion source temperature was maintained at 550 °C. The pressure of the nebulising and auxiliary gases was set at 55 psi (379.225 kPa; 1 psi = 6.895 kPa). The DP was set at 60/-60 V, and the CE was set at 35/-35 eV. The nebulising gas was nitrogen. The gas in question was nitrogen. The subsequent section will address the processes of data analysis and processing. The database of compounds in XLK capsule was constructed by compiling the relevant literature on the chemical components of Radix et Rhizoma Pinelliae, Radix Longan, Radix et Rhizoma Serpentis, Radix et Rhizoma Verticillatae, Radix Astragali, Radix et Rhizoma Ginseng, Radix et Rhizoma Pinelliae, Radix Angelica Sinensis, Radix et Rhizoma Polygoni Multiflori, and Radix et Rhizoma Danshen. The data collected by mass spectrometry were analysed using PeakView 1.2 software and compared with the information of the compounds in the database. The focus of the analysis was on the chromatographic retention time, quasi-molecular ion peaks and secondary characteristic fragments of the compound peaks. In addition, the analysis was combined with control products, a self-constructed database, the PubChem database and the unique mass spectrometry cleavage pattern of the compounds. This was done to analyse the chemical compositions of XLK capsule and the genus of the source herbs.

**Table S1 XLK prototype component identification**

| No. | Name | Found At RT (min) | Formula | Adduct | Found At Mass (Da) | Error (ppm) | MS/MS | source | TRUE |
| --- | --- | --- | --- | --- | --- | --- | --- | --- | --- |
| 1 | valine | 0.78 | C_5_H_11_NO_2_ | [M+H]^+^ | 118.0862 | -0.20 | 118.0866, 58.0654 | dg | TRUE |
| 2 | quinic acid | 0.80 | C_7_H_12_O_6_ | [M+H]^-^ | 191.0561 | 0.00 | 191.0554, 127.0388, 85.0288 | hq | TRUE |
| 3 | sucrose | 0.82 | C_12_H_22_O_11_ | [M+H]^-^ | 341.1094 | 1.30 | 341.1095, 179.0562, 161.0462 | auxiliary material | TRUE |
| 4 | pipecolic acid | 0.95 | C_6_H_11_NO_2_ | [M+H]^+^ | 130.0862 | -0.80 | 130.0876, 84.0817 | lk, dg | TRUE |
| 5 | uridine | 1.32 | C_9_H_12_N_2_O_6_ | [M+H]^+^ | 245.0770 | 0.70 | 245.1135, 113.0335 | lk | TRUE |
| 6 | pyroglutamic acid | 1.47 | C_5_H_7_NO_3_ | [M+H]^-^ | 128.0351 | -1.50 | 128.0335, 82.0302 | lk, dg, yj | TRUE |
| 7 | succinic acid | 1.65 | C_4_H_6_O_4_ | [M+H]^-^ | 117.0191 | -1.80 | 117.0180, 73.0287 | sm, hq | TRUE |
| 8 | gallic acid | 2.04 | C_7_H_6_O_5_ | [M+H]^-^ | 169.0143 | 0.60 | 169.0143, 125.0243 | lk, bzl, sm | TRUE |
| 9 | protocatechuic acid | 4.40 | C_7_H_6_O_4_ | [M+H]^-^ | 153.0194 | 0.40 | 153.0210, 109.0295, 91.0174 | ds, lk | TRUE |
| 10 | danshensu | 4.45 | C_9_H_10_O_5_ | [M+H]^-^ | 197.0457 | 0.80 | 179.0349, 135.0443, 123.0439, 72.9924 | ds | TRUE |
| 11 | feruloylputrescine | 5.40 | C_14_H_20_N_2_O_3_ | [M+H]^+^ | 265.1551 | 1.70 | 265.1550, 217.1305, 162.1151, 161.1067 | lk | TRUE |
| 13 | dihydrocaffeic acid | 5.52 | C_9_H_10_O_4_ | [M+H]^-^ | 181.0506 | 0.00 | 181.0504, 163.0379, 135.0422, 119.0496 | ds | TRUE |
| 14 | 4-hydroxybenzoic acid | 5.73 | C_7_H_6_O_3_ | [M+H]^-^ | 137.0244 | 0.10 | 137.0240, 93.0341 | sm, bzl | TRUE |
| 17 | 4-hydroxycoumarin | 6.38 | C_9_H_6_O_3_ | [M+H]^+^ | 163.0391 | 0.60 | 163.0397, 145.0277, 135.0440, 89.0383 | dg | TRUE |
| 18 | quercetin 3,4'-diglucoside | 6.53 | C_27_H_30_O_17_ | [M+H]^+^ | 627.1557 | 0.10 | 627.2504, 465.1012, 303.0500 | lk | TRUE |
| 19 | vanillin | 6.60 | C_8_H_8_O_3_ | [M+H]^-^ | 151.0400 | -0.50 | 151.0389, 136.0181, 108.0233 | sm, bzl | TRUE |
| 20 | esculetin | 6.62 | C_9_H_6_O_4_ | [M+H]^-^ | 177.0195 | 0.90 | 177.0182, 133.0323, 105.0345 | hq | TRUE |
| 23 | vicenin-2 | 7.10 | C_27_H_30_O_15_ | [M+H]^-^ | 593.1535 | 3.90 | 593.1543, 503.1215, 473.1107, 383.0791, 353.0680 | bzl | TRUE |
| 24 | 3-*p*-coumaroylquinic acid | 7.17 | C_16_H_18_O_8_ | [M+H]^-^ | 337.0936 | 2.00 | 337.0969, 191.0575, 119.0508, 93.0347 | lk, hs | TRUE |
| 25 | riboflavin | 7.18 | C_17_H_20_N_4_O_6_ | [M+H]^+^ | 377.1458 | 0.70 | 377.1444, 243.0874 | lk,hq | TRUE |
| 26 | isochlorogenic acid C | 7.30 | C_25_H_24_O_12_ | [M+H]^+^ | 517.1345 | 0.90 | 499.1209, 337.0901, 163.0385, 135.0431 | xl | TRUE |
| 27 | 3-*O*-feruloylquinic acid | 7.58 | C_17_H_20_O_9_ | [M+H]^-^ | 367.1043 | 2.30 | 367.1110, 191.0559, 173.0453, 93.0341 | lk | TRUE |
| 29 | isochondrodendrine | 7.72 | C_36_H_38_N_2_O_6_ | [M+H]^+^ | 595.2810 | 1.30 | 595.2775, 564.2337, 298.1416 | lhtg | TRUE |
| 30 | 4'-hydroxyacetophenone | 7.76 | C_8_H_8_O_2_ | [M+H]^-^ | 135.0451 | -0.20 | 135.0450, 93.0338 | dg | TRUE |
| 31 | 4-hydroxycinnamic acid | 7.79 | C_9_H_8_O_3_ | [M+H]^-^ | 163.0401 | 0.30 | 163.0420, 119.0509, 93.0339 | lk, bzl, dg, hs | TRUE |
| 32 | 3,5,6(8),7,4'-pentahydroxyflavanone-*O*-glucuronide | 7.98 | C_21_H_20_O_13_ | [M+H]^+^ | 481.0981 | 1.00 | 481.2011, 463.1752, 305.0645, 287.0538, 169.0124 | bzl | TRUE |
| 34 | jaceosidin-7-*O*-glucoside | 8.21 | C_23_H_24_O_12_ | [M+H]^-^ | 491.1218 | 4.60 | 491.1256, 283.0638, | xl | TRUE |
| 35 | calycosin 7-*O*-glucoside | 8.24 | C_22_H_22_O_10_ | [M+H]^+^ | 447.1290 | 1.10 | 285,0753, 253.0484 | hq | TRUE |
| 36 | vitexin | 8.25 | C_21_H_20_O_10_ | [M+H]^+^ | 433.1133 | 0.90 | 433.1132, 313.0702, 283.0594 | bzl | TRUE |
| 37 | lonicerin | 8.28 | C_27_H_30_O_15_ | [M+H]^+^ | 595.1665 | 1.30 | 595,1641, 449.1063, 287.0546, 153.0174 | xl | TRUE |
| 38 | isoacteoside | 8.31 | C_29_H_36_O_15_ | [M+H]^-^ | 623.2008 | 4.20 | 623.2024, 461.1671, 161.0243 | bzl | TRUE |
| 40 | hyperoside | 8.39 | C_21_H_20_O_12_ | [M+H]^-^ | 463.0901 | 4.10 | 463.0902, 301.0368 | lk, xl, sm |  |
| 41 | quercetin | 8.41 | C_15_H_10_O_7_ | [M+H]^+^ | 303.0509 | 3.30 | 303.0493, 257.0444, 229.0492, 165.0188 | lk, xl | TRUE |
| 42 | luteolin-7-*O*-glucoside | 8.47 | C_21_H_20_O_11_ | [M+H]^+^ | 449.1052 | -5.88 | 449.1052, 287.0549, 153.0165, 135.0411 | bzl | TRUE |
| 43 | scutellarin | 8.47 | C_21_H_18_O_12_ | [M+H]^+^ | 463.0884 | 2.80 | 463.0852, 287.0546, 145.0277, 119.0492 | bzl | TRUE |
| 45 | acteoside | 8.52 | C_29_H_36_O_15_ | [M+H]^-^ | 623.2010 | 4.50 | 623.2060, 461.1704, 315.1135,161.0256 | bzl | TRUE |
| 47 | apigenin-7-*O*-glucoside | 8.54 | C_21_H_20_O_10_ | [M+H]^+^ | 433.1133 | 0.90 | 433.1098, 271.0596, 153.0173, 119.0501 | bzl, xl | TRUE |
| 48 | genistein | 8.55 | C_15_H_10_O_5_ | [M+H]^+^ | 271.0608 | 2.70 | 271.0593, 153.0172, 145.0264, 119.0486 | sm, hq | TRUE |
| 49 | carthamidin-7-*O*-glucuronide | 8.70 | C_21_H_20_O_12_ | [M+H]^-^ | 463.0901 | 4.10 | 463.0918, 287.0578 | bzl |  |
| 50 | isorhamnetin-3-*O*-nehesperidine | 8.89 | C_28_H_32_O_16_ | [M+H]^-^ | 623.1652 | 5.60 | 623.2025, 577.1592, 269.0467 | lk | TRUE |
| 51 | salvianolic acid D | 8.94 | C_20_H_18_O_10_ | [M+H]^-^ | 417.0838 | 2.60 | 417.0887, 197.0472, 175.0414, 135.0455 | ds | TRUE |
| 52 | isoscutellarein-7-*O*-glucuronide | 8.95 | C_21_H_18_O_12_ | [M+H]^-^ | 461.0744 | 3.90 | 461.0788, 285.0420 | bzl | TRUE |
| 53 | isochlorogenic acid A | 8.96 | C_25_H_24_O_12_ | [M+H]^+^ | 517.1346 | 1.00 | 499.1200, 163.0381, 145.0272, 135.0424 | xl | TRUE |
| 55 | 6-hydroxyluteolin | 9.07 | C_15_H_10_O_7_ | [M+H]^+^ | 303.0506 | 2.20 | 303.0482, 169.0137, 137.0220, 123.0069 | bzl | TRUE |
| 56 | luteolin-4'-*O*-glucoside | 9.16 | C_21_H_20_O_11_ | [M+H]^+^ | 449.1078 | -0.20 | 449.1046, 287.0538, 135.0434 | lk | TRUE |
| 57 | genistin | 9.21 | C_21_H_20_O_10_ | [M+H]^+^ | 433.1133 | 0.80 | 433.1098, 271.0596, 153.0173, 119.0501 | hq | TRUE |
| 58 | luteolin-7-*O*-glucuronide | 9.24 | C_21_H_18_O_12_ | [M+H]^-^ | 461.0750 | 5.30 | 461.0739, 285.0418, 133.0292 | bzl, xl | TRUE |
| 59 | 8-methoxynaringenin-7-*O*-glucuronide | 9.25 | C_22_H_22_O_12_ | [M+H]^-^ | 477.1061 | 4.70 | 477.1085, 431.1020, 301.0743, 269.0478 | bzl | TRUE |
| 60 | 6-methoxynaringenin | 9.27 | C_16_H_14_O_6_ | [M+H]^+^ | 303.0866 | 1.00 | 303.0496, 183.0301, 147.0422 | bzl | TRUE |
| 61 | apigenin-7-*O*-glucuronide | 9.31 | C_21_H_18_O_11_ | [M+H]^+^ | 447.0928 | 1.40 | 447.0907, 271.0601, 153.0183 | bzl | TRUE |
| 62 | isochlorogenic acid B | 9.36 | C_25_H_24_O_12_ | [M+H]^+^ | 517.1345 | 0.90 | 499.1246, 319.0826, 163.0392, 135.0430 | xl | TRUE |
| 64 | hispidulin-7-*O*-glucoside | 9.41 | C_22_H_22_O_11_ | [M+H]^+^ | 463.1239 | 0.90 | 463.0848, 301.0697, 287.0529, 168.0051 | xl | TRUE |
| 65 | hispidulin-7-*O*-glucuronide | 9.42 | C_22_H_20_O_12_ | [M+H]^+^ | 477.1034 | 1.30 | 477.1000, 301.0702, 287.0566,147.0422 | bzl | TRUE |
| 66 | solanigroside P | 9.46 | C_39_H_63_NO_12_ | [M+H]^+^ | 738.4424 | 0.10 | 738.4383, 556.3704, 412.3234, 394.3087 | lk | TRUE |
| 67 | rosmarinic acid | 9.50 | C_18_H_16_O_8_ | [M+H]^-^ | 359.0785 | 3.40 | 359.0788, 197.0469, 161.0246, 135.0451 | ds | TRUE |
| 68 | 7-hydroxycoumarin | 9.50 | C_9_H_6_O_3_ | [M+H]^+^ | 163.0392 | 1.30 | 163.0383, 145.0655, 135.0431, 89.0393 | dg, xl | TRUE |
| 69 | 6-methoxynaringenin-7-*O*-glucuronide | 9.57 | C_22_H_22_O_12_ | [M+H]^-^ | 477.1049 | 2.30 | 477.1012, 301.0716, 175.0248 | bzl | TRUE |
| 70 | 8-methoxynaringenin | 9.58 | C_16_H_14_O_6_ | [M+H]^+^ | 303.0869 | 1.80 | 303.0490, 257.0505, 183.0271, 147.0408 | bzl | TRUE |
| 71 | jaranol | 9.59 | C_17_H_14_O_6_ | [M+H]^+^ | 315.0880 | 5.40 | 315.0846, 285.0393, 257.0435 | hq | TRUE |
| 72 | isosalvianolic acid A | 9.62 | C_26_H_22_O_10_ | [M+H]^-^ | 493.1164 | 4.80 | 493.1170, 313.0732, 295.0631, 185.0252 | ds | TRUE |
| 75 | solasonine | 9.72 | C_45_H_73_NO_16_ | [M+H]^+^ | 884.5008 | 0.60 | 884.4956, 738.4443, 576.3886, 414.3333, 147.0640 | lk | TRUE |
| 79 | ikonnikoside | 9.92 | C_21_H_18_O_12_ | [M+H]^+^ | 463.0885 | 3.10 | 463.0851, 287.0546, 145.0276, 119.0489 | bzl | TRUE |
| 80 | calycosin 7-(6'-acetylglucoside) | 9.94 | C_24_H_24_O_11_ | [M+H]^+^ | 489.1396 | 1.00 | 285.0749, 253.0480 | hq | TRUE |
| 81 | scutellarein | 9.99 | C_15_H_10_O_6_ | [M+H]^+^ | 287.0562 | 4.00 | 287.0553, 169.0126, 119.0493 | bzl | TRUE |
| 82 | ginsenoside Rg_1_ | 10.05 | C_42_H_72_O_14_ | [HCOOH-H]^-^ | 845.4945 | 6.10 | 845.4979, 799.4941, 637.4343, 475.3796 | hs | TRUE |
| 83 | chrysin-5-methylether | 10.06 | C_16_H_12_O_4_ | [M+H]^+^ | 269.0815 | 2.60 | 269.0789, 253.0479, 241.0499, 139.0047 | bzl | TRUE |
| 84 | senkyunolide F | 10.06 | C_12_H_14_O_3_ | [M+H]^+^ | 207.1020 | 1.98 | 207.0991, 189.0925, 161.0936, 91.0547 | dg | TRUE |
| 85 | salvianolic acid B | 10.06 | C_36_H_30_O_16_ | [M+H]^-^ | 717.1520 | 8.21 | 717.1520, 519.0966, 339.0530, 321.0429 | ds | TRUE |
| 86 | ononin | 10.07 | C_22_H_22_O_9_ | [M+H]^+^ | 431.1344 | 1.80 | 269.0800, 253.0475, 237.0535 | hq | TRUE |
| 87 | baicalin | 10.08 | C_21_H_18_O_11_ | [M+H]^+^ | 447.0932 | 2.20 | 447.0911, 271.0603, 253.0492, 103.0541 | bzl, hq | TRUE |
| 89 | senkyunolide K | 10.22 | C_12_H_16_O_3_ | [M+H]^+^ | 209.1173 | 0.50 | 191.0688, 163.0730, 91.0531 | dg | TRUE |
| 90 | isoscutellarein | 10.25 | C_15_H_10_O_6_ | [M+H]^+^ | 287.0563 | 4.40 | 287.0547, 169.0134, 119.0497 | bzl | TRUE |
| 94 | salvianolic acid A | 10.48 | C_26_H_22_O_10_ | [M+H]^-^ | 493.1168 | 5.60 | 493.1177, 313.0740, 295.0626, 185.0251, 135.0453 | ds | TRUE |
| 95 | salvianolic acid C | 10.50 | C_26_H_20_O_10_ | [M+H]^-^ | 491.1007 | 4.70 | 491.1011, 311.0598, 293.0487, 135.0456 | ds | TRUE |
| 96 | tetrahydrocurcumin | 10.56 | C_21_H_24_O_6_ | [M+H]^+^ | 373.1662 | 4.40 | 373.1641, 177.0904, 151.0752, 137.0598 | yj | TRUE |
| 97 | monomethyl Lithospermate | 10.73 | C_28_H_24_O_12_ | [M+H]^-^ | 551.1216 | 3.80 | 511.1269, 353.0688, 321.0441, 293.0484 | ds | TRUE |
| 98 | 9'''-methyl salvianolate B | 10.74 | C_37_H_32_O_16_ | [M+H]^-^ | 731.1661 | 6.00 | 731.1651, 533.1126, 353.0684, 309.0798 | ds | TRUE |
| 99 | 8,2'-dihydroxy-7,4'-dimethoxyisoflavan | 10.77 | C_17_H_18_O_5_ | [M+H]^+^ | 303.1235 | 2.70 | 303.0483, 167.0691, 123.0433 | hq | TRUE |
| 101 | isomucronulatol-7-*O*-β-*D*-glucoside | 10.81 | C_23_H_28_O_10_ | [M+H]^-^ | 463.1628 | 4.00 | 463.1686, 301.1102, 287.0604 | hq | TRUE |
| 102 | 8-hydroxyluteolin | 10.82 | C_15_H_10_O_7_ | [M+H]^+^ | 303.0507 | 2.40 | 303.0497, 167.0702, 153.0172, 137.0219 | bzl | TRUE |
| 103 | calycosin | 10.91 | C_16_H_12_O_5_ | [M+H]^-^ | 283.0626 | 5.00 | 283.0630, 268.0392, 239.0367, 211.0414 | hq, dg |  |
| 104 | wogonoside | 10.93 | C_22_H_20_O_11_ | [M+H]^-^ | 459.0956 | 5.00 | 459.0999, 283.0637, 268.0401, 240.0451 | bzl, hq |  |
| 105 | isosalvianolic acid C | 11.01 | C_26_H_20_O_10_ | [M+H]^-^ | 491.1009 | 5.20 | 491.0982, 311.0559, 293.0464, 135.0447 | ds | TRUE |
| 107 | dihydrocurcumin | 11.20 | C_21_H_22_O_6_ | [M+H]^+^ | 371.1490 | 0.20 | 371.1149, 285.0724, 217.0847, 137.0611 | yj | TRUE |
| 108 | dicoumaroyl Spemidine | 11.40 | C_25_H_31_N_3_O_4_ | [M+H]^+^ | 438.2386 | -0.40 | 438.2321, 292.1974, 204.1012, 147.0440 | lk | TRUE |
| 110 | 6"-*O*-acetylononin | 11.76 | C_24_H_24_O_10_ | [M+H]^+^ | 473.1448 | 1.10 | 473.1459, 269.0811, 253.0475, 237.0548 | hq | TRUE |
| 111 | cyclocanthoside E | 11.79 | C_41_H_70_O_14_ | [M+H]^+^ | 787.4842 | 0.50 | 787.4792, 619.4120, 457.3677 | hq | TRUE |
| 112 | apigenin | 11.80 | C_15_H_10_O_5_ | [M+H]^+^ | 271.0612 | 4.00 | 271.0607, 153.0182, 145.0281, 119.0494 | sm, bzl, xl, hq | TRUE |
| 113 | tigogenin-glucoside | 11.90 | C_33_H_54_O_8_ | [M+H]^+^ | 579.3896 | 0.70 | 579.3881, 417.3349, 273.2207 | lk | TRUE |
| 114 | timosaponin AIII | 11.91 | C_39_H_64_O_13_ | [M+H]^+^ | 741.4417 | -0.30 | 741.4415, 579.3883, 417.3373, 255.2110 | lk | TRUE |
| 115 | ginsenoside A_1_ | 11.92 | C_42_H_72_O_14_ | [HCOOH-H]^-^ | 845.4972 | 9.30 | 845.4995, 799.4950, 653.4379, 491.3882, 475.3823 | hs | TRUE |
| 116 | salvianolic acid F | 12.16 | C_17_H_14_O_6_ | [M+H]- | 313.0729 | 3.60 | 313.0721, 269.0472, 161.0248, 133.0298 | ds | TRUE |
| 117 | ginsenoside Rg_2_ | 12.37 | C_42_H_72_O_13_ | [HCOOH-H]^-^ | 829.5032 | 10.60 | 829.5051, 783.4991, 637.4434, 475.3849 | hs | TRUE |
| 119 | baicalein | 12.54 | C_15_H_10_O_5_ | [M+H]^+^ | 271.0612 | 4.10 | 271.0596, 169.0131, 102.0535, | bzl, hq | TRUE |
| 121 | 13*R*-14*R*-hydroxy-anhydrideof 16*R*-cryptotanshinone | 12.55 | C_19_H_22_O_5_ | [M+H]^-^ | 329.1404 | 2.80 | 329.2355, 241.1610 | ds | TRUE |
| 122 | sedanolide | 12.65 | C_12_H_18_O_2_ | [M+H]^+^ | 195.1383 | 1.80 | 149.1335, 93.0707 | dg | TRUE |
| 125 | pinocembrin | 12.76 | C_15_H_12_O_4_ | [M+H]^-^ | 255.0668 | 2.10 | 255.0697, 135.0070, 119.0483 | bzl, lk | TRUE |
| 131 | butylidenephthalide | 13.19 | C_12_H_12_O_2_ | [M+H]^+^ | 189.0912 | 1.00 | 171.0806, 153.0738, 105.0324 | dg | TRUE |
| 132 | syringaresinol | 13.26 | C_22_H_26_O_8_ | [M+H]^-^ | 417.1565 | 2.50 | 371.1515, 151.0746 | xl, hq | TRUE |
| 133 | 15,16-dihydrotanshindiol B | 13.54 | C_18_H_18_O_5_ | [M+H]^-^ | 313.1086 | 1.60 | 313.1848, 298.1599, 283.0265, 269.0496 | ds | TRUE |
| 134 | veratramine | 13.55 | C_27_H_39_NO_2_ | [M+H]^+^ | 410.3060 | 1.50 | 410.3055, 392.2940, 267.1738 | lk | TRUE |
| 135 | marmesin | 13.55 | C_14_H_14_O_4_ | [M+H]^+^ | 247.0975 | 4.00 | 247.1143, 229.0852, 187.0388 | dg | TRUE |
| 136 | isoastragaloside II | 13.58 | C_43_H_70_O_15_ | [M+H]^+^ | 827.4797 | 1.10 | 827.4682, 629.3983, 455.3474, 175.0593, 143.1067 | hq | TRUE |
| 137 | barbatellarine C | 13.60 | C_30_H_37_NO_10_ | [M+H]^+^ | 572.2501 | 1.80 | 572.2658, 124.0392 | bzl | TRUE |
| 138 | isomucronulatol | 13.65 | C_17_H_18_O_5_ | [M+H]^+^ | 303.1237 | 3.20 | 167.0698, 133.0644, 123.0433 | hq | TRUE |
| 140 | scutehenanine H | 13.80 | C_26_H_31_NO_7_ | [M+H]^+^ | 470.2179 | 1.30 | 479.2197, 124.0386 | bzl | TRUE |
| 141 | soyasaponin I | 13.94 | C_48_H_78_O_18_ | [M+H]^+^ | 943.5273 | 1.30 | 943.5166, 797.4644, 635.4142, 599.3905, 423.3595 | hq | TRUE |
| 142 | methylnissolin | 13.95 | C_17_H_16_O_5_ | [M+H]^+^ | 301.1076 | 2.00 | 301.1028, 271.0582, 191.0697, 167.0705 | hq | TRUE |
| 144 | cyclocephaloside II | 14.08 | C_43_H_70_O_15_ | [M+H]^+^ | 827.4804 | 1.90 | 827.4612, 629.4019, 455.3501, 175.0581 | hq | TRUE |
| 146 | scutebarbatine L | 14.25 | C_32_H_36_N_2_O_8_ | [M+H]^+^ | 577.2555 | 1.80 | 577.2532, 454.2177, 124.0386 | bzl | TRUE |
| 147 | wogonin | 14.28 | C_16_H_12_O_5_ | [M+H]^-^ | 283.0626 | 4.90 | 283.0643, 268.0407, 239.0369, 224.0495 | bzl, hq |  |
| 148 | 3-hydroxymethylenetanshinquinone | 14.30 | C_18_H_14_O_4_ | [M+H]^-^ | 293.0831 | 4.00 | 293.0820, 265.0874, 249.0547, 221.0608 | ds | TRUE |
| 150 | chrysin | 14.40 | C_15_H_10_O_4_ | [M+H]^+^ | 255.0661 | 3.60 | 255.0652, 153.0172, 103.0527 | bzl | TRUE |
| 152 | astragaloside II | 14.48 | C_43_H_70_O_15_ | [M+H]^+^ | 827.4806 | 2.20 | 827.4716, 611.3860, 437.3383, 175.0586, 143.1061 | hq | TRUE |
| 153 | salvianonol | 14.70 | C_18_H_20_O_4_ | [M+H]^+^ | 301.1444 | 3.20 | 301.1429, 283.1319, 265.1212, 241.1230 | ds | TRUE |
| 155 | nortanshinone | 14.78 | C_17_H_12_O_4_ | [M+H]^+^ | 281.0813 | 1.70 | 281.0810, 253.0857, 235.0763, 207.0809 | ds | TRUE |
| 156 | tanshinone IIB | 14.88 | C_19_H_18_O_4_ | [M+H]^+^ | 311.1290 | 4.00 | 311.1277, 293.1166, 283.1316, 265.1214 | ds | TRUE |
| 158 | astragaloside I | 15.12 | C_45_H_72_O_16_ | [M+H]^+^ | 869.4915 | 2.50 | 869.4614, 689.4589, 455.3512, 143.1061 | hq |  |
| 159 | scutebarbatine X | 15.14 | C_34_H_38_N_2_O_10_ | [M+H]^+^ | 635.2609 | 1.50 | 635.2587, 575.2371, 124.0392, 106.0270 | bzl | TRUE |
| 160 | scutelinquanine B | 15.26 | C_32_H_38_N_2_O_9_ | [M+H]^+^ | 595.2657 | 1.10 | 595.2610, 124.0395, 106.0271 | bzl | TRUE |
| 164 | scutelinquanine A | 15.44 | C_28_H_35_NO_8_ | [M+H]^+^ | 514.2442 | 1.20 | 514.2408, 124.0386, 106.0254, | bzl | TRUE |
| 165 | isoastragaloside I | 15.57 | C_45_H_72_O_16_ | [M+H]^+^ | 869.4911 | 2.00 | 869.4846, 671.4207, 455.3494, 143.1053 | hq |  |
| 167 | tanshinone VI | 15.63 | C_18_H_16_O_4_ | [M+H]^+^ | 297.1130 | 2.80 | 297.1133, 279.1004, 261.0916, 233.0968 | ds | TRUE |
| 168 | trijuganone C | 15.66 | C_20_H_20_O_5_ | [M+H]^+^ | 341.1390 | 2.00 | 341.1370, 281.1173, 263.1065, 235.1112 | ds | TRUE |
| 169 | scutebarbatine A | 15.74 | C_32_H_34_N_2_O_7_ | [M+H]^+^ | 559.2450 | 1.90 | 559.2412, 436.2068, 313.1775, 124.0387 | bzl | TRUE |
| 170 | curcolone | 15.90 | C_15_H_18_O_3_ | [M+H]^+^ | 247.1339 | 4.00 | 247.1318, 229.1225, 201.1272, 139.0387 | yj | TRUE |
| 171 | curzeone | 15.90 | C_15_H_16_O_2_ | [M+H]^+^ | 229.1226 | 1.30 | 229.1217, 211.1100, 201.1257, 153.0690 | yj | TRUE |
| 173 | scutelinquanine C | 16.11 | C_31_H_39_NO_8_ | [M+H]^+^ | 554.2754 | 0.90 | 554.2678, 436.2092, 313.1779, 124.0383 | bzl | TRUE |
| 174 | tanshinone V | 16.13 | C_19_H_22_O_4_ | [M+H]^+^ | 315.1602 | 3.50 | 315.1543, 297.1477, 253.1586 | ds | TRUE |
| 175 | 13*S*-hydroxy-9*Z*,11*E*-octadecadienoic acid | 16.45 | C_18_H_32_O_3_ | [M+H]^+^ | 297.2430 | 1.90 | 297.1482, 279.1935, 253.1573, 251.1009 | hq | TRUE |
| 176 | scutebarbatine K | 16.51 | C_28_H_33_NO_7_ | [M+H]^+^ | 496.2334 | 0.80 | 496.2340, 124.0388 | bzl | TRUE |
| 177 | tanshinaldehyde | 16.61 | C_19_H_18_O_4_ | [M+H]^+^ | 311.1284 | 1.90 | 311.1260, 293.1248, 275.2038 | ds | TRUE |
| 178 | methylenetanshinquinone | 16.79 | C_18_H_14_O_3_ | [M+H]^+^ | 279.1027 | 4.00 | 279.1007, 261.0907, 233.0957, 205.1009 | ds | TRUE |
| 179 | OA-Glur A | 16.81 | C_36_H_56_O_9_ | [M+H]^-^ | 631.3886 | 5.50 | 631.3907, 455.3518 | hs | TRUE |
| 180 | tanshialdehyde | 16.85 | C_19_H_16_O_4_ | [M+H]^+^ | 309.1121 | 0.00 | 309.1100, 281.1159, 263.1055, 235.1115 | ds | TRUE |
| 181 | *Z*-ligustilide | 17.20 | C_12_H_14_O_2_ | [M+H]^+^ | 191.1073 | 3.20 | 191.1064, 173.0959, 161.0606, 145.1021 | dg | TRUE |
| 183 | neocryptotanshinone | 17.32 | C_19_H_22_O_4_ | [M+H]^+^ | 315.1602 | 3.60 | 315.1589, 297.1485, 279.1379, 268.1088 | ds | TRUE |
| 184 | neotanshinone B | 17.32 | C_18_H_16_O_3_ | [M+H]^+^ | 281.1182 | 3.50 | 281.1168, 263.1061, 235.1114, 220.0880 | ds | TRUE |
| 185 | scutebarbatine W | 17.41 | C_33_H_37_NO_8_ | [M+H]^+^ | 576.2574 | -3.11 | 576.2574, 454.2193, 124.0389, 105.0316 | bzl | TRUE |
| 186 | acetytastragaloside I | 17.50 | C_47_H_74_O_17_ | [M+H]^+^ | 911.5022 | 2.60 | 911.4906, 713.4230, 455.3539, 259.0797, 143.1056 | hq | TRUE |
| 187 | methyl tanshinonate | 17.53 | C_20_H_18_O_5_ | [M+H]^+^ | 339.1236 | 2.70 | 339.1192, 279.1007, 261.0907, 233.0962 | ds | TRUE |
| 188 | isoagroastragaloside V | 17.56 | C_43_H_72_O_14_ | [M+H]^-^ | 811.4923 | 9.10 | 811.4965, 765.4854, 603.4332 | hq | TRUE |
| 189 | agroastragaloside V | 17.77 | C_43_H_72_O_14_ | [M+H]^-^ | 811.4924 | 9.30 | 811.4944, 765.4851, 603.4343 | hq | TRUE |
| 192 | prionoid E | 18.26 | C_20_H_22_O_4_ | [M+H]^+^ | 327.1605 | 4.50 | 327.0780, 267.1357, 249.1279, 207.0803 | ds | TRUE |
| 193 | cryptotanshinone | 18.60 | C_19_H_20_O_3_ | [M+H]^+^ | 297.1500 | 5.00 | 297.1482, 282.1241, 268.1089, 254.0932 | ds | TRUE |
| 194 | tanshinone I | 18.74 | C_18_H_12_O_3_ | [M+H]^+^ | 277.0864 | 1.80 | 277.0862, 249.0912, 234.0678, 178.0778 | ds | TRUE |
| 196 | dihydrotanshinone I | 19.51 | C_18_H_14_O_3_ | [M+H]^+^ | 279.1027 | 3.90 | 279.0962, 261.0904, 233.0958, 205.1011 | ds | TRUE |
| 197 | dehydrotanshinone IIA | 19.66 | C_19_H_16_O_3_ | [M+H]^+^ | 293.1182 | 3.40 | 293.1174, 278.0926, 275.1054, 247.1111 | ds | TRUE |
| 198 | cryptoacetalide | 19.75 | C_18_H_22_O_3_ | [M+H]^+^ | 287.1654 | 4.20 | 287.1633, 269.1527, 254.1296, 239.1060 | ds | TRUE |
| 199 | salviolone | 19.75 | C_18_H_20_O_2_ | [M+H]^+^ | 269.1540 | 1.50 | 269.1560, 254.1267, 239.1046 | ds | TRUE |
| 200 | angelicide | 19.86 | C_24_H_28_O_4_ | [M+H]^+^ | 381.2071 | 2.80 | 381.2048, 335.1997, 191.1064 | dg | TRUE |
| 201 | tanshinlactone | 20.03 | C_17_H_12_O_3_ | [M+H]^+^ | 265.0859 | -0.30 | 265.0857, 250.0631, 237.0894 | ds | TRUE |
| 203 | dehydromiltirone | 20.20 | C_19_H_20_O_2_ | [M+H]^+^ | 281.1550 | 5.00 | 281.1544, 266.1301, 263.1441, 253.1594 | ds | TRUE |
| 204 | scutebata G | 20.27 | C_40_H_41_NO_9_ | [M+H]^+^ | 680.2855 | 0.10 | 680.2850, 124.0392 | bzl | TRUE |
| 205 | levistolide A | 20.39 | C_24_H_28_O_4_ | [M+H]^+^ | 381.2066 | 1.60 | 191.1068, 173.0957, 149.0606, 135.0447 | dg | TRUE |
| 206 | tanshinone IIA | 20.57 | C_19_H_18_O_3_ | [M+H]^+^ | 295.1319 | -3.29 | 295.1319, 280.1083, 277.1211, 249.1256 | ds | TRUE |
| 207 | dihydro crytotanshinone | 20.95 | C_19_H_22_O_3_ | [M+H]^+^ | 299.1650 | 2.80 | 299.1623, 281.1528, 253.1579, 223.1113 | ds | TRUE |
| 208 | miltrione | 21.07 | C_19_H_22_O_2_ | [M+H]^+^ | 283.1706 | 4.80 | 283.1689, 268.1453, 265.1586, 240.1145 | ds | TRUE |
| 210 | oleamide | 23.32 | C_18_H_35_NO | [M+H]^+^ | 282.2801 | 3.50 | 282.2788, 265.2520, 97.1006, 69.0700 | lk | TRUE |
| 211 | linoleic acid | 23.59 | C_18_H_32_O_2_ | [M+H]^-^ | 279.2341 | 4.10 | 279.2335, 134.8958 | bzl | TRUE |
| 212 | pheophorbide A | 23.95 | C_35_H_36_N_4_O_5_ | [M+H]^+^ | 593.2767 | 1.40 | 593.2729, 533.2522, 461.2296 | lk | TRUE |

**note：**dg：*Angelica sinensis(Oliv.) Diels*；hq：*Astragalus membranaceus (Fisch.) Bunge*；lk：*Solanum nigrum L*；yj：*Curcuma aromatica Salisb*；sm：*Duchesnea indica (Andrews) Focke*；bzl：*Scutellaria barbata D. Don*；ds：*Salvia miltiorrhiza Bunge*；lhtj：*Cyclea racemosa Oliv*；hs：*Panax ginseng C.A.Mey*；xl：*Saussurea involucrate (Kar. & Kir.) Sch. Bip*

**Table S2 Key Components of XLK forImproving CIF (Top 30)**

| **NO.** | **source** | **CAS** | | **English Name** | **Molecular**  **Formula** | **Molecular**  **Weight** |
| --- | --- | --- | --- | --- | --- | --- |
| 1 | *Angelica sinensis（Oliv.）Diels* | | 1076-38-6 | 4-hydroxycoumarin | C_9_H_6_O_3_ | 163.04 |
| 2 | *Scutellaria barbata D.Don* | | 480-40-0 | chrysin | C_15_H_10_O_4_ | 255.07 |
| 3 | *Scutellaria barbata D.Don* | | 491-67-8 | baicalein | C_15_H_10_O_5_ | 271.06 |
| 4 | *Scutellaria barbata D.Don* | | 33554-47-1 | chrysin-5-methylether | C_16_H_12_O_4_ | 269.08 |
| 5 | *Astragalus membranaceus (Fisch.) Bunge* | | 3301-49-3 | jaranol | C_17_H_14_O_6_ | 315.09 |
| 6 | *Duchesnea indica (Andrews) Focke* | | 520-36-5 | apigenin | C_15_H_10_O_5_ | 271.06 |
| 7 | *Solanum nigrum L.* | | 117-39-5 | quercetin | C_15_H_10_O_7_ | 303.05 |
| 8 | *Scutellaria barbata D.Don* | | 61303-13-7 | isoacteoside | C_29_H_36_O_15_ | 623.20 |
| 9 | *Scutellaria barbata D.Don* | | 60-33-3 | linoleic acid | C_18_H_32_O_2_ | 279.23 |
| 10 | *Scutellaria barbata D.Don* | | 529-53-3 | scutellarein | C_15_H_10_O_6_ | 287.06 |
| 11 | *Scutellaria barbata D.Don* | | 36508-73-5 | 6-hydroxyluteolin | C_15_H_10_O_7_ | 303.05 |
| 12 | *Scutellaria barbata D.Don* | | 4136-37-2 | 8-hydroxyluteolin | C_15_H_10_O_7_ | 303.05 |
| 13 | *Duchesnea indica (Andrews) Focke* | | 446-72-0 | genistein | C_15_H_10_O_5_ | 271.06 |
| 14 | *Scutellaria barbata D.Don* | | 480-39-7 | pinocembrin | C_15_H_12_O_4_ | 255.07 |
| 15 | *Curcuma aromatica Salisb.* | | 36062-04-1 | tetrahydrocurcumin | C_21_H_24_O_6_ | 373.16 |
| 16 | *Scutellaria barbata D.Don* | | 142796-21-2 | scutebarbatine K | C_28_H_33_NO_7_ | 496.23 |
| 17 | *Astragalus membranaceus (Fisch.) Bunge* | | 110596-84-2 | 8,2'-dihydroxy-  7,4'-dimethoxyisoflavan | C_17_H_18_O_5_ | 303.12 |
| 18 | *Astragalus membranaceus (Fisch.) Bunge* | | 110596-85-3 | isomucronulatol | C_17_H_18_O_5_ | 303.12 |
| 19 | *Astragalus membranaceus (Fisch.) Bunge* | | 110596-86-4 | methylnissolin | C_17_H_16_O_5_ | 301.11 |
| 20 | *Salvia miltiorrhiza Bunge* | | 110596-87-5 | prionoid E | C_20_H_22_O_4_ | 327.16 |
| 21 | *Salvia miltiorrhiza Bunge* | | 110596-88-6 | tanshinaldehyde | C_19_H_18_O_4_ | 311.13 |
| 22 | *Curcuma aromatica Salisb.* | | 36062-05-2 | dihydrocurcumin | C_21_H_22_O_6_ | 371.15 |
| 23 | *Salvia miltiorrhiza Bunge* | | 110596-89-7 | salvianonol | C_18_H_20_O_4_ | 301.14 |
| 24 | *Angelica sinensis（Oliv.）Diels* | | 6066-49-5 | butylidenephthalide | C_12_H_12_O_2_ | 189.09 |
| 25 | *Scutellaria barbata D.Don* | | 110596-90-0 | 6-methoxynaringenin | C_16_H_14_O_6_ | 303.09 |
| 26 | *Angelica sinensis（Oliv.）Diels* | | 486-35-1 | 7-hydroxycoumarin | C_9_H_6_O_3_ | 163.04 |
| 27 | *Solanum nigrum L.* | | 110596-91-1 | feruloylputrescine | C_14_H_20_N_2_O_3_ | 265.15 |
| 28 | *Salvia miltiorrhiza Bunge* | | 568-73-0 | tanshinone IIB | C_19_H_18_O_4_ | 311.13 |
| 29 | *Salvia miltiorrhiza Bunge* | | 110596-92-2 | cryptoacetalide | C_18_H_22_O_3_ | 287.16 |
| 30 | *Salvia miltiorrhiza Bunge* | | 568-74-9 | tanshinone VI | C_18_H_16_O_4_ | 297.11 |

**Table S3 Regulation of Core Targets by XLK Active Components**

| Protein Name | UniProt ID | Gene Name | Corresponding Components |
| --- | --- | --- | --- |
| Mechanistic Target of Rapamycin | Q9JLN9 | MTOR | 3-Hydroxy-9,10-dimethoxypterocarpan, 7,2'-Dihydroxy-3',4'-dimethoxyisoflavan, Scutebarbatine K, 8,2'-Dihydroxy-7,4'-dimethoxyisoflavan, Pinocembrin |
| Serine/Threonine-Protein Kinase AKT | P31750 | AKT1 | Scutellarein, 4-Hydroxycoumarin, Jaranol, 7-Hydroxycoumarin, 8-Hydroxyluteolin, 7,2'-Dihydroxy-3',4'-dimethoxyisoflavan, 6-Hydroxyluteolin, Quercetin, 8,2'-Dihydroxy-7,4'-dimethoxyisoflavan, Isoacteoside, Apigenin |
| Epidermal Growth Factor Receptor | Q01279 | EGFR | 6-Hydroxyluteolin, Scutebarbatine K, Scutellarein, 8-Hydroxyluteolin, Baicalein, Chrysin, Isoacteoside, 4-Hydroxycoumarin, Cryptoacetalide, Prionoid E, Tanshinone IIB, Tanshinone VI, Astragalus Isoflavonoid, Jaranol, Feruloylputrescine, Quercetin, Apigenin, Dihydrocurcumin, Genistein |
| Insulin Receptor | Q6PDK2 | INSR | Quercetin, Baicalein, 6-Hydroxyluteolin, Chrysin, 4-Hydroxycoumarin, 8-Hydroxyluteolin, Tanshinone IIB, Jaranol, Chrysin-5-methylether |
| Caspase-3 | P70677 | CASP3 | 7,2'-Dihydroxy-3',4'-dimethoxyisoflavan, 3-Hydroxy-9,10-dimethoxypterocarpan, Tanshinaldehyde |
| Estrogen Receptor 1 | P19785 | ESR1 | Linoleic Acid, Scutebarbatine K, 6-Methoxynaringenin, Baicalein, Butylidenephthalide, Chrysin-5-methylether, Genistein, Jaranol, Apigenin, Scutellarein, 4-Hydroxycoumarin, Tetrahydrocurcumin, Isoacteoside, 6-Hydroxyluteolin, Chrysin, Pinocembrin, Dihydrocurcumin, 3-Hydroxy-9,10-dimethoxypterocarpan |
